# Synthesis of Primary Amines via Reductive Amination of Aldehydes and Ketones Over a Ni‐Doped MFM‐300(Cr) Catalyst

**DOI:** 10.1002/advs.202508892

**Published:** 2025-10-24

**Authors:** Wenyuan Huang, Bing An, Zeyu Chen, Yu Han, Yinlin Chen, Jiangnan Li, Xue Han, Shaojun Xu, Danielle Crawshaw, Evan Tillotson, Bing Han, Sarah J. Haigh, Christopher M. A. Parlett, Luke Keenan, Svemir Rudić, Yongqiang Cheng, Ben F. Spencer, Martin Schröder, Sihai Yang

**Affiliations:** ^1^ College of Chemistry and Molecular Engineering Beijing National Laboratory for Molecular Sciences Peking University Beijing 100871 China; ^2^ Department of Chemistry University of Manchester Manchester M13 9PL UK; ^3^ College of Chemistry Beijing Normal University Beijing 100875 China; ^4^ Department of Chemical Engineering University of Manchester Manchester M13 9PL UK; ^5^ Department of Materials University of Manchester Manchester M13 9PL UK; ^6^ Diamond Light Source Harwell Science Campus Oxfordshire OX11 0DE UK; ^7^ UK Catalysis Hub Rutherford Appleton Laboratory Oxfordshire OX11 0FA UK; ^8^ ISIS Facility STFC Rutherford Appleton Laboratory Oxfordshire OX11 0AF UK; ^9^ Neutron Scattering Division, Neutron Sciences Directorate Oak Ridge National Laboratory Oak Ridge TN TN 37830 USA

**Keywords:** catalysis, MOF, neutron scattering, reductive amination, structure

## Abstract

The development of earth‐abundant metal‐based catalysts is an important goal for the synthesis of fine chemicals. Here, an active nickel catalyst supported on a robust metal–organic framework, MFM‐300(Cr), is reported which shows an exceptional performance for reductive amination, a reaction that has long been dominated by noble metals. Ni/MFM‐300(Cr) promotes the synthesis of 38 primary amines via reductive amination of their parent carbonyl compounds, including biomass‐derived aldehydes and ketones, using NH_3_ in the presence of H_2_ operating under relatively mild conditions (5 bar and 160 °C). X‐ray absorption spectroscopy confirms the formation of mixtures of metallic Ni^0^ and Ni^n+^ active sites, while in situ inelastic neutron scattering, coupled with modeling, reveals details of the mechanism of catalysis involving the formation of N‐benzyl‐1‐phenylmethanediamine (BPDI) as an intermediate species in the generation of benzylamine. Cooperativity between Ni sites and MFM‐300(Cr) creates an optimal microenvironment for the efficient activation of carbonyl compounds and the selective production of primary amines using a non‐precious metal‐based catalyst.

## Graphical abstract

Reductive amination of aldehydes and ketones to primary amines with high selectivity and yield can be achieved over Ni‐doped MFM‐300(Cr) in the presence of NH_3_ and H_2_. In situ inelastic neutron scattering, coupled with modeling, reveals that cooperativity between Ni active sites and MFM‐300(Cr) affords an optimal microenvironment for the selective conversion.

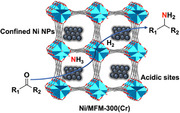

## 1 Introduction

**Figure 1:**
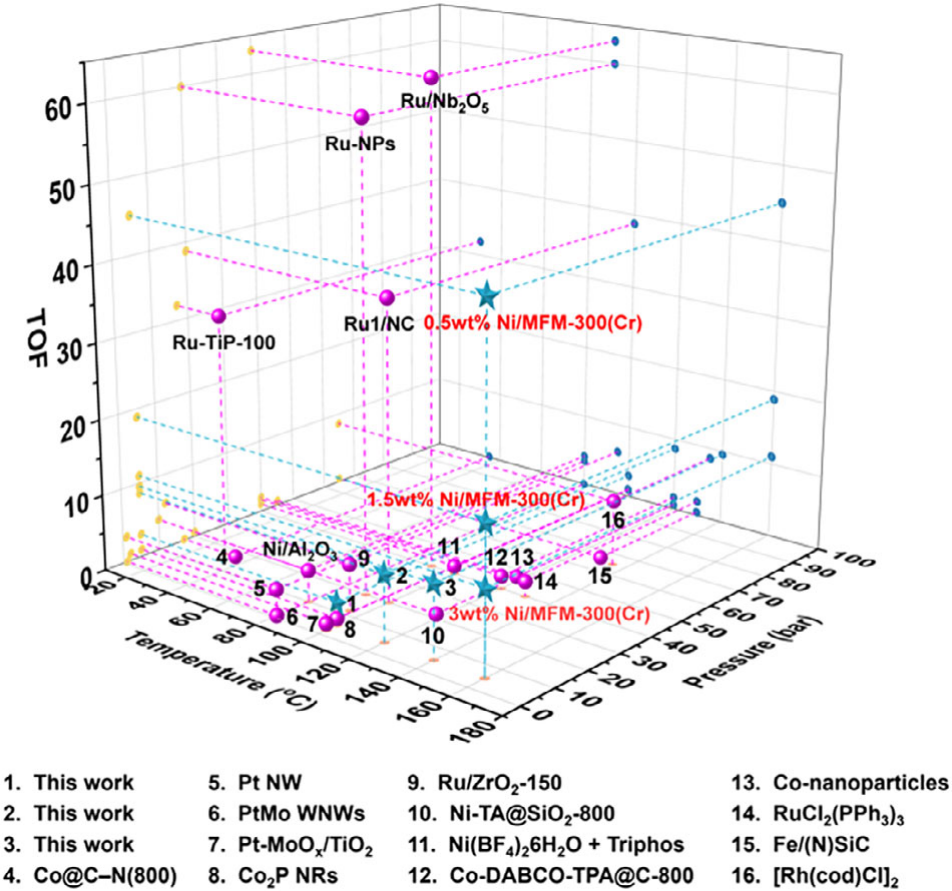
Comparison of the performance of state‐of‐the‐art catalysts for reductive amination of benzaldehyde with NH_3_ and H_2_. Comparison of TOF (turnover frequency) versus reaction temperature and pressure. Full data are given in Table S1 (Supporting Information). Note: Data points 1–3 (blue stars) correspond to 3 wt.% Ni/MFM‐300(Cr) tested in this work at 100, 120, and 140 °C, respectively. The blue stars correspond to 0.5 wt.% Ni/MFM‐300(Cr), 1.5 wt.% Ni/MFM‐300(Cr) and 3 wt.% Ni/MFM‐300(Cr) and were tested in this work at 160 °C.

Primary amines are used widely to produce pharmaceuticals, fine chemicals, polymers, agrochemicals, and materials.^[^ ^1^, ^2^, ^3^ ^]^ Reductive amination of carbonyl compounds, especially biomass‐derived aldehydes and ketones, using NH_3_ and H_2_ as a nitrogen resource and reductant, respectively, represents a promising strategy to synthesise primary amines using renewable resources. A variety of homogeneous and heterogeneous catalysts have been investigated, and state‐of‐the‐art catalysts are generally based upon noble metals (**Figure** **1**).^[^ ^4^, ^5^, ^6^, ^7^, ^8^, ^9^, ^10^, ^11^, ^12^ ^]^ Heterogeneous catalysts show advantages such as ease of recovery, reusability, and stability, and various catalytic systems based upon zeolites, Al_2_O_3_, ZrO_2_ and supported Co,^[^ ^1^ ^]^ Ni,^[^ ^3^ ^]^ Ru,^[^ ^8^ ^]^ Pt,^[^ ^11^ ^]^ and Pd^[^ ^12^ ^]^ species have been developed. However, the poor selectivity to primary amines remains a major challenge due to possible side reactions.^[^ ^8^, ^9^, ^10^ ^]^ Furthermore, the catalyst must survive the presence of an alkaline environment formed by NH_3_ and amines. Therefore, powerful drivers exist to exploit new alkaline‐stable catalysts based upon earth‐abundant metals for the selective synthesis of primary amines.

Metal–organic framework (MOF) materials possess designable pore structures, which can provide a versatile platform to confine and activate substrates via specific adsorption behavior and binding.^[^ ^13^, ^14^, ^15^ ^]^ The effect of host‐guest interactions has been studied extensively in zeolites,^[^ ^16^, ^17^ ^]^ supramolecular cages,^[^ ^18^, ^19^, ^20^ ^]^ and MOFs.^[^ ^21^, ^22^ ^]^ Due to their stability and structural diversity, MOFs show excellent potential as supports or co‐catalysts for a range of reactions, such as CO_2_ reduction,^[^ ^23^, ^24^, ^25^ ^]^ hydrogen/oxygen evolution,^[^ ^26^, ^27^ ^]^ degradation of organic pollutions^[^ ^28^ ^]^ and selective hydrogenation of olefins.^[^ ^29^ ^]^ However, to date, little success has been achieved in the construction of C‐N bonds through reductive amination over MOF‐based catalysts.

Here, we report the selective synthesis of primary amines via reductive amination of ketones and aldehydes (38 substrates) over alkaline‐stable Ni/MFM‐300(Cr) using NH_3_ and H_2_ as nitrogen source and reductant, respectively, under relatively mild conditions (5 bar and 160 °C). Notably, Ni/MFM‐300(Cr) exhibits an exceptional activity and selectivity for benzylamine, with a turnover frequency (TOF) of 45.1 (Figure 1; Table S1, Supporting Information). This performance outperforms all state‐of‐the‐art non‐noble Ni, Co, and Fe‐based metal catalysts and compares favorably with leading noble‐metal Ru‐based catalysts (e.g., Ru^1^/NC^[^ ^8^ ^]^). Although our system requires relatively high temperature (160 °C) compared with Ni/Al_2_O_3_^[^ ^3^ ^]^ (operating at ≤80 °C and ≤10 bar H_2_), it gives excellent yields and selectivity across a broad range of substrates, including sterically hindered and functionalized substrates, and demonstrates superior catalyst reusability without metal leaching. Synchrotron X‐ray absorption spectroscopy (XAS) and inelastic neutron scattering (INS) confirm that strong host‐guest interactions and acidic sites collectively afford efficient activation of adsorbed substrates and the formation of N‐benzyl‐1‐phenylmethanediamine (BPDI) as an intermediate species, thereby driving the selective synthesis of primary amines. While previous studies have proposed the formation of such intermediates based upon theoretical or indirect experimental evidence, to the best of our knowledge, this work provides the first direct evidence for the formation of the unstable intermediate BPDI under catalytic conditions using neutron spectroscopy. This assignment would benefit from further validation using direct structural or complementary spectroscopic data via isotopic labeling, solid‐state NMR, or *operando* XAS techniques.

## 2 Results and Discussion

MFM‐300(Cr),^[^ ^22^ ^]^ [Cr_2_(OH)_2_(L)]_∞_ (H_4_L = biphenyl‐3,3′,5,5′‐tetracarboxylic acid), was selected due to its high NH_3_ adsorption capacity (Figure S1, Supporting Information) and high stability. Owing to its activity toward hydrogenation,^[^ ^3^, ^6^ ^]^ Ni was selected as a dopant to generate active sites for catalytic reductive amination. Samples of catalyst Ni/MFM‐300(Cr) with different loadings of Ni (0.5–5.0 wt.%) were prepared using a double solvent method^[^ ^30^ ^]^ from desolvated MFM‐300(Cr) via addition of an aqueous solution of NiBr_2_ followed by reduction using NaBH_4_ (see Experimental Section, Supporting Information). Various other catalysts, based upon MOFs and metal oxides (e.g., Cr_2_O_3_, Al_2_O_3_, ZrO_2_), were also prepared for comparison.

**Figure 2:**
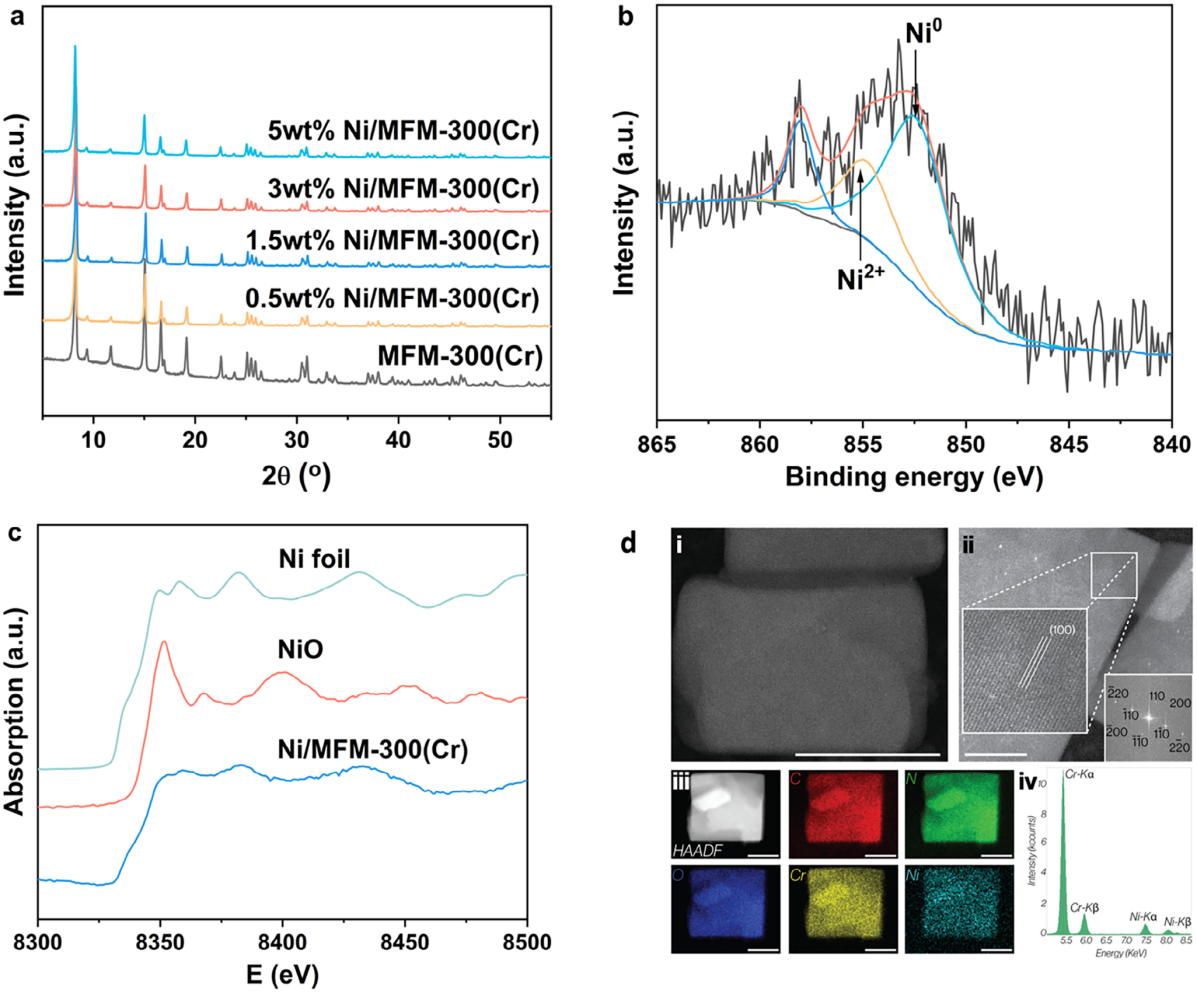
Characterisation of Ni/MFM‐300(Cr). a) PXRD patterns of MFM‐300(Cr) and as‐prepared Ni/MFM‐300(Cr) at different Ni loadings. b) XPS spectra of Ni 2p_3/2_ of Ni/MFM‐300(Cr). c) XANES spectra at the Ni K‐edge of Ni foil, NiO, and Ni/MFM‐300(Cr). d) i) High‐angle annular dark field (HAADF) scanning transmission electron microscopy (STEM) of Ni/MFM‐300(Cr) at low magnification; ii) high‐resolution STEM of Ni/MFM‐300(Cr) with fast Fourier transform (FFT) in inset showing lattice resolution along the [001] direction. iii) Survey HAADF‐STEM image and energy dispersive X‐ray spectroscopy (EDS) elemental maps of carbon, nitrogen, oxygen, chromium, and nickel for Ni/MFM‐300(Cr). iv) Integrated EDS spectrum summed from the survey image in iii). The scale bars for i), ii) and iii) are 100, 50, and 200 nm, respectively. The reflections shown in the FFT were indexed as MFM‐300(Cr) using CrystalDiffract simulation software.^[^ ^34^ ^]^

Powder X‐ray diffraction (PXRD) confirmed retention of the structure of MFM‐300(Cr) upon introduction of Ni, and no additional Bragg peak on incorporation of Ni was observed (**Figure** **2**a). The mass loading of Ni was measured by inductively coupled plasma optical emission spectrometry (ICP‐OES) (Table S2, Supporting Information). N_2_ adsorption measurements confirm a decrease of specific surface area, for example, from 1146 m^2^ g^−1^ for MFM‐300(Cr) to 728 m^2^ g^−1^ for 3 wt.% Ni/MFM‐300(Cr) (Figure S3, Supporting Information). X‐ray photoelectron spectroscopy (XPS) identified both metallic Ni^0^ and Ni^2+^ sites to be present in the catalyst in a ratio of ≈2.5:1, respectively (Figure 2b). Likewise, Ni K‐edge X‐ray absorption near‐edge structure (XANES) analysis of Ni/MFM‐300(Cr), using Ni foil and NiO as reference materials, shows features of both Ni(0) and Ni(II) (Figure 2c),^[^ ^31^ ^]^ consistent with the XPS results. We propose that Ni^0^ species play a crucial role in the activation of H_2_, a step that initiates the reductive pathway. In parallel, Ni^2^⁺ centres ‐ possibly anchored within the MOF pore environment ‐ may act as Lewis acidic sites that stabilise the imine intermediate and promote selective hydrogenation. The coexistence of these two oxidation states may, therefore, enable a cooperative bifunctional mechanism, enhancing the overall activity and selectivity. This hypothesis is supported by the high conversion rates and reusability observed, as well as recent mechanistic reports on dual‐site Ni catalysts.^[^ ^32^, ^33^ ^]^

The location and distribution of Ni sites within MFM‐300(Cr) were investigated by scanning transmission electron microscopy (STEM; Figure 2d) and EDS elemental mapping combined with N_2_ sorption analyses. The absence of large Ni aggregates in low‐magnification TEM images, along with the uniform elemental dispersion, suggests that Ni sites are well‐dispersed. Furthermore, the slight reduction in surface area and pore volume after Ni incorporation supports the partial occupation of MOF pores.

**Figure 3:**
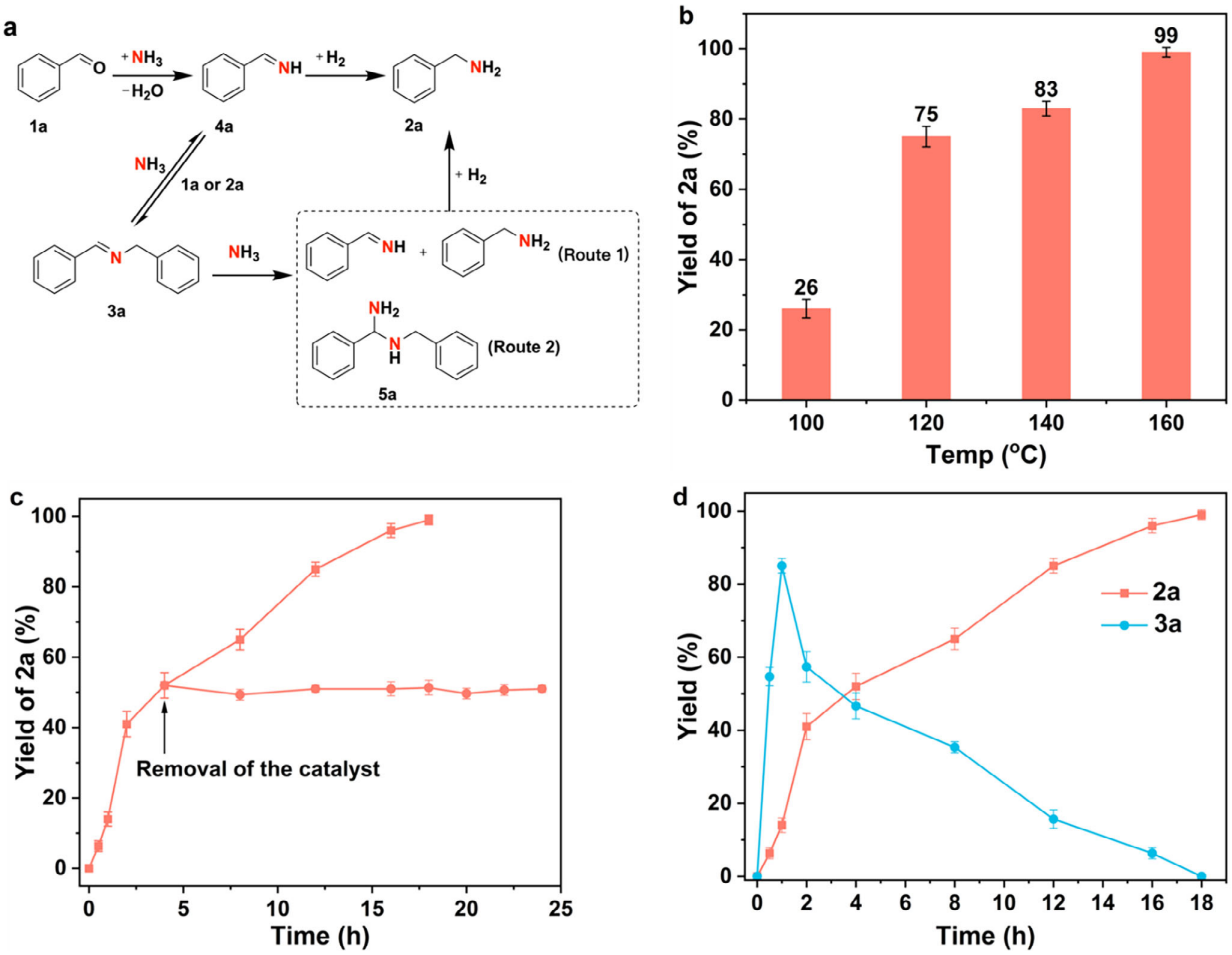
Catalytic performance. a) Main reaction pathway of reductive amination of benzaldehyde (**1a**) to benzylamine (**2a**). b) The yield of **2a** as a function of temperature over Ni/MFM‐300(Cr). c) Leaching test and d) Time course for reductive amination of **1a** over Ni/MFM‐300(Cr). Reaction conditions: 3 wt.% Ni/MFM‐300(M) (10 mg catalyst, 0.005 mmol Ni), 1 mmol benzaldehyde, NH_3_ in MeOH (1 mL, 7 mmol, 7 mol L^−1^), 1 mL MeOH, 5 bar H_2_, 160 °C, 18 h. Yields were determined by GC using n‐dodecane as an internal standard.

The reaction conditions for reductive amination over Ni/MFM‐300(Cr) were optimised using benzaldehyde and NH_3_ for the synthesis of benzylamine; the proposed main reaction pathway is shown in **Figure** **3**a. Screening of solvents confirmed a notable preference for protic solvents in facilitating the formation of primary amines, and the use of MeOH can effectively suppress the formation of undesired by‐products such as benzyl alcohol (Figure S4, Supporting Information). Other factors influencing the yield of primary amine were also investigated, including the amount of Ni, NH_3_, pressure of H_2_ and reaction temperature (Figures 3b; S5–S7, Supporting Information). In summary, the optimised conditions for the highest conversion and yield of amine utilise 3 wt.% Ni/MFM‐300(Cr), 7.0 mmol NH_3_ and 2.0 mL of MeOH at 160 °C and 5 bar H_2_. Although the 0.5 wt.% sample exhibited the highest TOF, its conversion was only around 69% (Figure S7, Supporting Information), and so the 3 wt.% sample catalyst was chosen for all subsequent experiments. Unless noted otherwise, Ni/MFM‐300(Cr) in this study refers therefore to 3 wt.% Ni loading.

The catalytic activities of different catalysts have been explored under optimised conditions using benzaldehyde as the substrate (**Table** **1**). Significantly, Ni/MFM‐300(Cr) shows an excellent yield of 99% for the target product of benzylamine (**2a**) on full conversion. In contrast, MFM‐300(Cr) and NiO do not generate benzylamine, demonstrating the critical role of Ni(0) sites. Interestingly, Ni/MFM‐300(Al) also shows full conversion of benzaldehyde but a lower yield of benzylamine (90%), suggesting that the acidity of the [M─O(H)─M] (M ═ Cr, Al) moieties plays an important role in the activation of substrates. Ni/Cr_2_O_3_, Ni/Al_2_O_3,_ and Ni/ZrO_2_, prepared via the same method, showed notably lower yields of benzylamine (58%, 69% and 54%, respectively) compared with Ni/MFM‐300(Cr) (Table 1). Bulk Ni powder shows very poor activity (yield of 6% for benzylamine), but interestingly, a powdered mixture of Ni powder and MFM‐300(Cr) displays an increased yield of 35% (conversion is increased from 48% to 84%), indicating the key role of MFM‐300(Cr) in the activation of substrates likely via adsorption‐induced host‐guest interactions. Thus, these results confirm that the direct cooperation of Ni nanoparticles and MFM‐300(Cr) plays a vital role in the reductive amination. We have also performed a control experiment using BPI under identical catalytic conditions (160 °C, 5 bar H_2_) but without adding NH_3_. The Ni/MFM‐300(Cr) catalyst efficiently converts BPI to the corresponding secondary amine, dibenzylamine, in 91% yield. However, for reductive amination of benzaldehyde over Ni/MFM‐300(Cr) (Table 1) only BPI and primary amine (benzylamine) are detected, suggesting NH_3_ is important for the selectivity of this reaction.

**Table 1:** Reductive amination of benzaldehyde (**1a**) to benzylamine (**2a**) and **3a** using various catalysts.

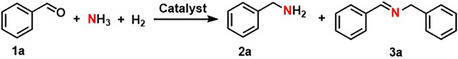				
Entry^a)^	Catalyst	Conversion [%]	Yield [%]	
			2a	3a
1	none	32	0	23
2	MFM‐300(Cr)	45	0	38
3	Ni/MFM‐300(Cr)	100	99	1
4	Ni/MFM‐300(Al)	100	90	10
5	Ni/ZrO_2_	100	54	41
6	Ni/Cr_2_O_3_	100	58	39
7	Ni/Al_2_O_3_	100	69	28
8	Ni powder	48	6	29
9	Ni powder + MFM‐300(Cr)	84	35	46
10	Ni/MFM‐300(Cr)‐D	87	58	17
11	NiO	68	0	47

^a)^ Reaction conditions: 3 wt.% Ni/MFM‐300(M) (10 mg catalyst, 0.005 mmol Ni), 1 mmol benzaldehyde, NH_3_ in MeOH (1 mL, 7 mmol, 7 mol L^−1^), 1 mL MeOH, 5 bar H_2_, 160 °C, 18 h. Yields were determined by GC using n‐dodecane as an internal standard. For all other catalysts listed in the table, the amount of Ni was fixed at 0.005 mmol. Ni/MFM‐300(Cr)‐D is the sample that is pre‐treated to partially collapse the pore structure of MFM‐300(Cr) at 350 °C.

The heterogeneous nature of this system has been studied by leaching experiments. Ni/MFM‐300(Cr) was removed by centrifugation when the yield of benzylamine reached 50%, and little further production of benzylamine was observed (Figure 3c). The concentrations of Ni and Cr in the filtrate were found to be negligible (below the detection limit of ICP), confirming the heterogeneous nature of this system. Furthermore, the catalyst can be retrieved readily from the reaction mixture by centrifugation with a recovery rate of >90%. The recovered catalyst can be reused for at least five cycles with consistent yields of benzylamine >98% (Figure S8, Supporting Information). The PXRD patterns of the fresh and used catalyst after five cycles of reaction confirm the retention of the structure of the MFM‐300(Cr) platform (Figure S9, Supporting Information).

**Figure 4:**
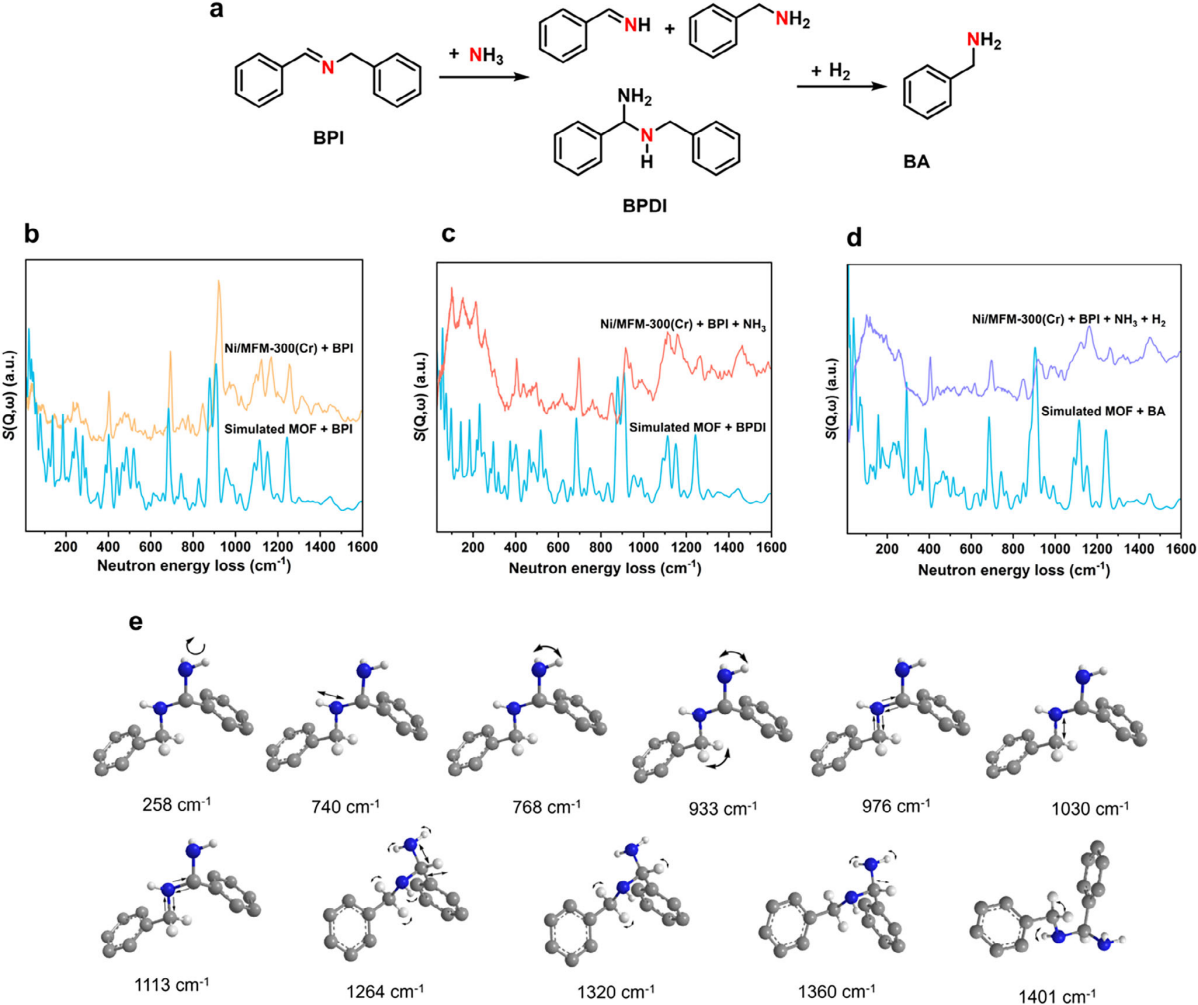
INS study of the reductive amination. a) Transformation of the Schiff base (BPI) to benzylamine (BA) via formation of BPDI. b) INS spectrum of adsorbed BPI in Ni/MFM‐300(Cr) and simulated spectrum. c) INS spectra of adsorbed BPI n Ni/MFM‐300(Cr) after reaction with NH_3_, and d) reaction with NH_3_ and H_2_. e) Illustration of selected vibrational modes of BPDI.

Following the progress of the reaction and the formation of products by GC, it was confirmed that benzaldehyde (**1a**) can be converted to the Schiff base N‐benzyl‐1‐phenylmethanimine (BPI) (**3a**) rapidly, followed by subsequent reduction to benzylamine (**2a**) (Figure 3d). Schiff base products can be hydrogenated directly to a secondary amine or transformed to the gem‐diamine BPDI, which can be converted by hydrogenation to benzylamine (**Figure** **4**a). As a result, the specific pathway taken by the Schiff base determines the selectivity of products.

In situ INS is a useful technique for probing the vibrational dynamics of hydrogen‐containing compounds, and was employed to elucidate the reaction mechanism. Though imine or gem‐diamines have been recognised widely as the key intermediates in reductive amination (Figure 4a), their presence in the reaction pathway has been poorly evidenced due to their relative instability. INS, coupled with density functional theory (DFT) calculations, enabled direct visualisation of various adsorption states, thus elucidating the reaction pathway. BPI is formed directly by the reaction of benzaldehyde and benzylamine (Figure 3a), and we sought to elucidate its subsequent reaction with NH_3_/H_2_ in the presence of Ni/MFM‐300(Cr). BPI was thus adsorbed into Ni/MFM‐300(Cr) to form BPI@ Ni/MFM‐300(Cr), the INS spectrum of which matches that from DFT simulations (Figure 4b). The peaks at low energy below 150 cm^−1^ corresponding to the lattice modes of BPI decrease significantly upon adsorption, suggesting that the BPI molecules are strongly adsorbed into the MOF, resulting in heavily hindered motion. The intensities of peaks at 1027 cm^−1^ (C_7_‐N stretching), 1202 cm^−1^ (C_7_‐H twisting), and 1376 cm^−1^ (C_9_‐H rocking) also decrease owing to adsorption of the C═N bond onto the catalyst.

NH_3_ was dosed into BPI@Ni/MFM‐300(Cr), and this was followed by reaction with H_2_ in the presence of NH_3_ leading to hydrogenation to give benzylamine (Figure 4). Catalytic conversion of adsorbed BPI with NH_3_ (at 120 °C for 1 h) was monitored for possible formation of imine intermediates, adducts of NH_3,_ and/or formation of BPDI, which are generally difficult to isolate and characterise due to their low stability. The INS spectrum of BPI@Ni/MFM‐300(Cr) changed significantly on reaction with NH_3_ with the new spectrum closely matching the DFT‐simulated spectrum of BPDI@Ni/MFM‐300(Cr) (Figure 4c). Representative peaks at 1113 cm^−1^ (N_8_‐C_9_‐N_16_), 1030 cm^−1^ (C_7_‐N_8_, C_9_‐N_8_, C_9_‐N_16_ stretching), 258 cm^−1^ (‐NH_2_ torsion), 740 cm^−1^ (N_8_‐H rocking), 768 cm^−1^ (N_16_‐H wagging), 933 cm^−1^ (N_16_‐H wagging coupled with C_7_‐H rocking), 1160 cm^−1^ (N_16_‐H twisting coupled with C_9_‐H rocking) and 1264 cm^−1^ (N_16_‐H twisting coupled with C_7_‐H twisting) are observed, confirming the formation of BPDI. Importantly, this represents the first observation of this unstable intermediate using neutron spectroscopy. Hydrogenation was then performed at 120 °C for 3 h under 5 bar of H_2_ (Figure 4d), and the characteristic peaks of BPDI at 768, 933, 1113, 1320 and 1360 cm^−1^ decreased with its consumption. New peaks appear at 464, 578, and 779 cm^−1^ corresponding to the C‐C out‐of‐plane wagging, C‐H twisting, and C─H wagging in the benzene ring, respectively. Additionally, the modes of C─C─C twisting located at 205 and 299 cm^−1^ (coupled with ─NH_2_ torsion) also decreased or disappeared, suggesting changes in the binding environment of the benzene ring. Notably, the peak shifts from 258 to 266 cm^−1^ (‐NH_2_ torsion), 1360 to 1436 cm^−1^ (C‐H wagging coupled with NH_2_ twisting) and 1401 to 1496 cm^−1^ (C‐H scissoring) indicate the formation of a new type of ‐CH_2_NH_2_ group. Overall, the INS peaks post‐hydrogenation are entirely consistent with the DFT‐simulated spectrum of benzylamine. Thus, the catalytic route to benzylamine from BPI can be proposed (Figure 3a, Route 2). Initially, BPDI is formed from the reaction of NH_3_ with BPI@Ni/MFM‐300(Cr), and subsequently, the active H^·^ species generated at the Ni sites react with BPDI to produce benzylamine under the optimised reaction conditions.

The scope of reductive amination over Ni/MFM‐300(Cr) was investigated using 38 aldehydes and ketones (**Table** **2**). The conversion of ketones is more challenging and generally requires more drastic reaction conditions or more active catalysts, but for many aldehydes and ketones the yields of the corresponding primary amines were greater than 90%. The position of substitution in 3‐ or 4‐methylbenzaldehyde has a negligible impact on the yield of the corresponding amines, and similar results were observed for 3‐ and 4‐methylacetophenone (Table 2). Halogenated aldehydes with fluoro‐, chloro‐ and bromo‐ groups can be transformed into the corresponding amines in high yield. Aliphatic aldehydes were also tested, and excellent yields of primary amines were obtained. Reductive amination of selected natural products and biomass derivatives has also been investigated over Ni/MFM‐300(Cr) and the corresponding primary amines obtained in high yield. For example, the conversion of biomass‐derived furfural and 5‐methyl furfural gave high yields (95–99%) of the primary amines.

**Table 2:** Yields of products from reductive amination reactions catalysed by Ni/MFM‐300(Cr).

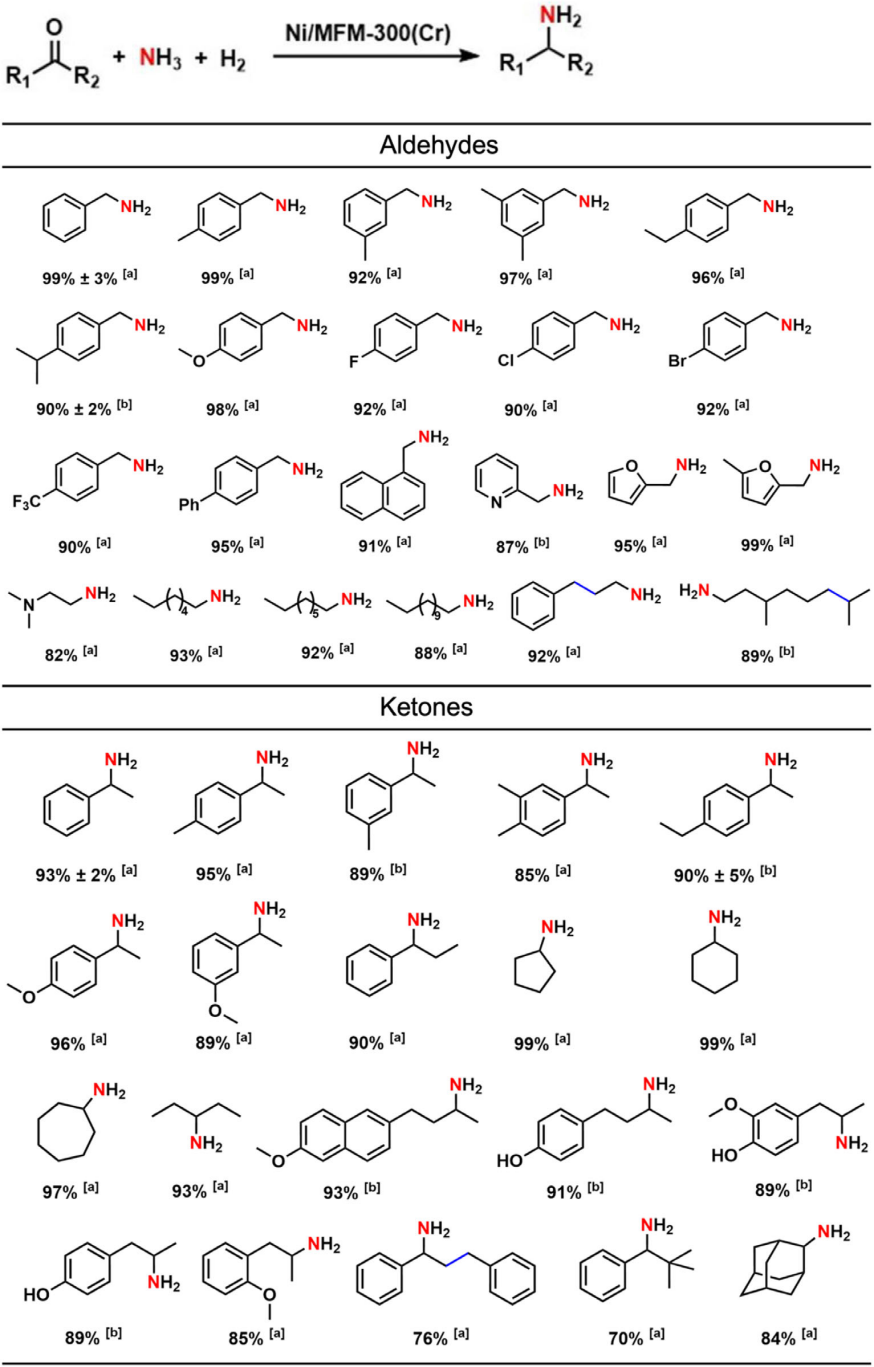

Reaction conditions: 3 wt.% Ni/MFM‐300(M) (10 mg catalyst, 0.005 mmol Ni), 1 mmol substrate, NH_3_ in MeOH (1 mL, 7 mmol, 7 mol L^−1^), 1 mL MeOH, 5 bar H_2_, 160 °C, 18 h. Yields are reported as either isolated yields after column chromatography (noted with [a]) or NMR yields determined by NMR using 1,3,5‐trimethoxybenzene as an internal standard (noted with [b]). Error margins (± standard deviation) represent results from triplicate experiments under identical conditions. It should be noted that reaction with cinnamaldehyde, chalcone and citronellal led to successful reductive amination, but with complete hydrogenation of the C═C double bond.

## 3 Conclusion

In summary, we have developed an active nickel‐based catalyst for the reductive amination of a wide range of aliphatic and aromatic carbonyl compounds in NH_3_/MeOH solution under mild conditions to afford primary amines. The synergy between active Ni(0) sites and confined adsorption of substrates in MFM‐300(Cr) collectively facilitates the activation of carbonyl compounds and results in excellent catalytic performance without the use of precious metals. The catalyst can be re‐used for five times consecutively without loss of activity, and in situ INS/DFT analysis confirmed the formation and stabilisation of BPDI as a key intermediate over Ni/MFM‐300(Cr).

## Conflict of Interest

The authors declare no conflict of interest.

## Supporting information



Supporting Information

## Data Availability

The data that support the findings of this study are available from the corresponding author upon reasonable request.
