# Supplementary material for: Synthesis of Primary Amines via Reductive Amination of Aldehydes and Ketones Over a Ni‐Doped MFM‐300(Cr) Catalyst
Source: Adv Sci (Weinh). 2025 Oct 24;12(46):e08892. doi: 10.1002/advs.202508892 (PMC12697848; doi:10.1002/advs.202508892)
Supplement: Supplementary file 1 — Supporting Information [file ADVS-12-e08892-s001.docx]

**Supplementary Information**

**Synthesis of primary amines *via* reductive amination of aldehydes and ketones over a Ni-doped MFM-300(Cr) catalyst**

Wenyuan Huang,^[a,b]^ Bing An,^[b]^ Zeyu Chen,^[b]^ Yu Han,^[b]^ Yinlin Chen,^[b]^ Jiangnan Li,^[a,b]^ Xue Han,^[b,c]^ Shaojun Xu,^[d]^ Danielle Crawshaw,^[b]^ Evan Tillotson,^[e]^ Bing Han,^[e]^ Sarah J. Haigh^[e]^, Christopher M. A. Parlett,^[d,f,g]^ Luke Keenan,^[f]^ Svemir Rudić,^[h]^ Yongqiang Cheng,^[i]^ Ben F. Spencer,^[e]^ Martin Schröder*^[b]^ and Sihai Yang*^[a,b]^

[a] Dr. W. Huang, Dr. J. Li and Prof. S. Yang
College of Chemistry and Molecular Engineering, Beijing National Laboratory for Molecular Sciences, Peking University, Beijing 100871 (China) Sihai.Yang@pku.edu.cn

[b] Dr. W. Huang, Dr. B. An, Z. Chen, Dr. Y. Han, Dr. Y. Chen, Dr. J. Li, D. Crawshaw, Prof. M. Schröder and Prof. S. Yang
Department of Chemistry, University of Manchester, Manchester, M13 9PL (UK) M.Schroder@manchester.ac.uk

[c] Prof. X. Han

College of Chemistry, Beijing Normal University, Beijing 100875 (China)

[d] Dr. S. Xu and Dr. C.M.A. Parlett
Department of Chemical Engineering, University of Manchester, Manchester, M13 9PL (UK)

[e] Dr. E. Tillotson, Dr. B. Han, Prof. S.J. Haigh and Dr. B.F. Spencer

Department of Materials, University of Manchester, Manchester, M13 9PL (UK)

[f] Dr. C.M.A. Parlett and Dr. L. Keenan

Diamond Light Source, Harwell Science Campus, Oxfordshire, OX11 0DE (UK)

[g] Dr. C.M.A. Parlett

UK Catalysis Hub, Rutherford Appleton Laboratory, Oxfordshire, OX11 0FA (UK)

[h] Dr. S. Rudić

ISIS Facility, STFC Rutherford Appleton Laboratory, Oxfordshire OX11 0AF (UK)

[i] Dr. Y. Cheng
Neutron Scattering Division, Neutron Sciences Directorate, Oak Ridge National Laboratory, Oak Ridge, TN (USA)

**1. Experimental Section**

**1.1 Materials**

All chemicals and reagents used in this study were purchased from Fischer Scientific or Sigma Aldrich and used as received without further purification.

**1.2 Catalyst preparation**

**1.2.1 Synthesis of MFM-300(Cr)**

MFM-300(Cr) was synthesised according to the literature method^[1]^. H_4_L (biphenyl-3,3’,5,5’-tetracarboxylic acid) (70 mg, 0.21 mmol), CrCl_3_·6H_2_O (200 mg, 0.75 mmol), deionised water (10 mL) and 1% HCl (1.5 mL) were mixed and transferred into a Teflon-lined stainless-steel autoclave, which was sealed and heated at 210 °C for 3 days. After cooling to room temperature, the resultant blue solid product was collected by centrifugation, washed with water and hot DMF several times, and dried at 60 °C under vacuum for 12 h. To remove DMF from within the pores, the as-synthesised powder was processed by exchanging the acetone using a Soxhlet extractor followed by drying at 130 °C overnight.

**1.2.2 Synthesis of Ni/MFM-300(Cr)**

Ni(II) salts were encapsulated into MFM-300(Cr) using a double solvent method^[2]^. Typically, 500 mg of desolvated MFM-300(Cr) was suspended in 40 mL of dry n-hexane as a hydrophobic solvent and the mixture sonicated for 10 mins until it becomes homogeneous. After stirring for 5 mins, 0.2 mL of aqueous NiBr_2_ solution of desired concentration was added dropwise as the hydrophilic solvent with vigorous stirring. The resulting solution was stirred for 5 h. The solid which settled to the bottom of the vial was collected and dried at 80 ^o^C under vacuum for 12 h. The reduction of the doped material was carried out using 0.6 M aqueous NaBH_4_ solution with vigorous stirring for 2 h. The reduced catalyst was collected by centrifugation and washed with MeOH several times and dried under vacuum at room temperature for 24 h. All the metal-loaded samples were prepared using the same method except for the addition of different support material and/or metal precursors.

**1.3 Characterisation**

**Powder X-ray diffraction (PXRD)** patterns were measured on a Phillips X’pert Modular Powder Diffractometer fitted with a copper K-α1 source (λ = 1.5406 Å). The PXRD patterns were measured between 2*θ* values of 4° and 55° with a step size of 0.0167° and 34.9 s per degree.

**N_2_ sorption isotherms** were measured on a Micromeritics 3-Flex gas sorption analyser at 77 K. Samples were acetone-exchanged for 24 h and activated at 170 °C for 12 h under dynamic vacuum prior to measurements.

**Elemental analysis and determination of metal content** were performed using a Thermo Scientific iCAP 6000 Series ICP spectrometer and a Thermo Scientific Flash 2000 organic elemental analyser. The preparation of solutions for elemental analysis were prepared as follows: an accurately weighed sample was added to a 12 mL Teflon-lined stainless-steel autoclave, and 750 μL of concentrated HNO_3_ and 2250 μL of concentrated HCl were added. The autoclave was sealed and heated at 145 °C for 12 h. On cooling to room temperature, the mixture was diluted with deionised water and transferred to a 10 mL volumetric flask. The final solution was used for elemental analysis.

**NMR spectroscopic measurement:** ^1^H NMR and ^13^C NMR spectra were collected at room temperature in DMSO-*d_6_* on Bruker B500 or B400 spectrometers.

**X-ray photoelectron spectroscopy (XPS)**. XPS measurements were carried out on a Kratos Axis Ultra Hybrid spectrometer using Al Kα X-ray radiation (1486.6 eV, 150 W at 15 kV bias); a charge neutraliser was used to replenish the electron supply at the surface. Survey spectra were measured using a pass energy of 80 eV and high-resolution core levels with a pass energy of 40 eV. Charge (binding energy scale) calibration was performed using C 1s at 284.8 eV, and data were analysed using CASAXPS (www.casaxps.com).

**X-ray absorption near edge structure (XANES)**. XANES at Ni K-edge were measured on the I20 beamline at the Diamond Light Source, Didcot, UK, employing non-monochromatic X-rays produced by a bent crystal polychromator and fast moving exit slits to enable a scan collection time of 4 s. Data collection spanned 200 eV before the edge and 1000 eV past it, with a step size of 0.3 eV; for energy calibration a Ni foil reference sample was used. Data processing was carried out using the Demeter open source software package (version 0.9.26) with XAS spectra processing (normalisation and background subtractions) and linear combination fitting conducted within the Athena programmes. Reference spectra of NiO standards were collected after dilution in boron nitride.

**Inelastic neutron scattering (INS)** spectra were recorded on the TOSCA spectrometer at the ISIS Facility at the STFC Rutherford Appleton Laboratory (UK), which is an indirect geometry crystal analyser instrument. TOSCA provides a wide dynamic range with high-resolution spectra in the 0-2000 cm^−1^ range. All INS spectra were collected after the sample was cooled and stabilized at temperatures below 15 K. The temperature during data collection was controlled by using a closed cycle refrigerator (CCR) cryostat (15 ± 0.1 K) and data collected at various points in the reaction.

In a typical experiment, the catalyst (~5 g) was loaded into a flow-type Inconel cell that can also be used as a static cell with all valves closed. The sample was heated at 120 °C (5 °C min^−1^ ramp rate) under He for 3 h to remove any remaining trace water/solvents before the experiment. N-Benzylidenebenzylamine (BPI, 1 g) dissolved in MeOH was injected into the cell at 25°C to allow adsorption of BPI adsorption into the catalyst. NH_3_/MeOH (1 mL) was then injected into the cell at 25 °C. Before data collection, the cell was flushed with dry He to remove weakly bound substrate and any excess solvent, and the sample cooled to < 15 K before data collection. H_2_ at 5 bar was dosed at 25 °C into the cell, which was then sealed and heated up to 160 °C for monitoring reaction over different times. After each reaction, the cell was quenched in liquid N_2_ and INS data collected to detect the presence of possible reaction intermediates. INS spectra of BPI adsorbed in Ni/MFM-300(Cr) were collected at 5 K.

**DFT Calculations and Modelling of the INS Spectra**: Periodic density functional theory (periodic-DFT) calculations were carried out using the plane wave pseudopotential method as implemented in the CASTEP code.^[3,4]^ Exchange and correlation were approximated using the Perdew-Burke-Ernzerhof (PBE) functional.^[5]^ Ultra-soft pseudopotentials were employed to account for the effects of core electrons. Tkatchenko-Scheffler dispersion correction^[6]^ was used for van der Waals interactions. Energy cutoff for plane-wave basis was 380 eV, and phonon frequencies were obtained by diagonalisation of dynamical matrices computed using finite displacement method. The atomic displacements in each mode that are part of the CASTEP output enable visualization of the modes to aid assignments and are also all that is required to generate the INS spectrum using the program OCLIMAX.^[7, 8]^

DFT calculations of the INS spectra for adsorbed BPI, BPDI and benzylamine (BA) were carried out, and used to identify the modes of vibrational features in the experimental INS spectra. The calculated INS spectrum shows the total transitions (up to 10 orders).

**High-angle annular dark-field scanning transmission electron microscopy (HAADF-STEM):** HAADF-STEM images and EDX elemental maps were collected on a Thermo Fisher Talos STEM (G2 80-200) equipped with high-angle annual dark-field (HAADF) detector and ChemiSTEM Super-X EDX detector, operating at 200 kV.

1.4 Reductive amination of carbonyl compounds with H_2_ in NH_3_/MeOH.

In a typical procedure, the catalyst [3 wt% Ni/MFM-300(M) (10mg catalyst, 0.005 mmol Ni)] was added to 1 mL of NH_3_/MeOH (7mmol, 7 mol/L), 1mL MeOH, and 1 mmol of carbonyl compound added into the reactor, which was then sealed. The reactor was flushed with N_2_ three times to remove air, and H_2_ (5 bar) was introduced and the cell heated at 160 °C for 18 h under stirring. When the reaction was complete, it was cooled to room temperature using an ice bath and degassed slowly to ambient pressure. The yield of resultant product was measured by GC or isolated by column chromatography.

**1.5 Leaching experiment**

A hot filtration test was performed to confirm that the catalyst does not form homogeneous sites during the reaction. When a 50% yield of benzylamine had been produced, the hot catalytic mixture was filtered to remove the catalyst. The filtrate was then tested under the same catalytic conditions described above and the formation of potential products analysed.

**2. Supplementary Figures**

**
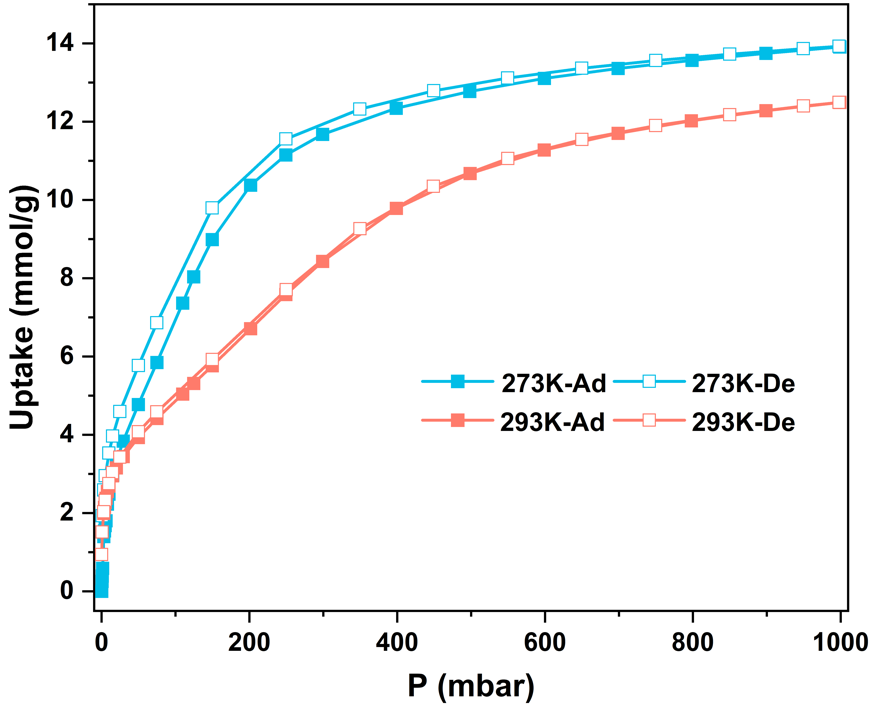
**

**Figure S1:** Adsorption-desorption isotherms for NH_3_ in MFM-300(Cr).

**
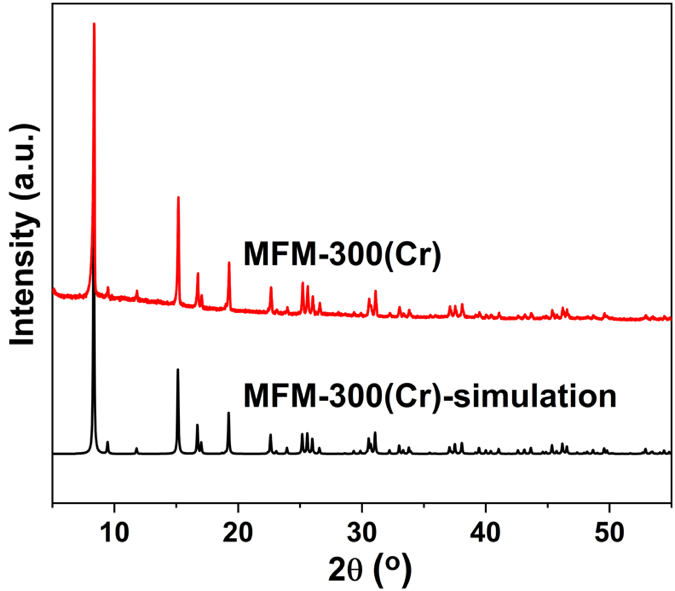
**

**Figure S2:** PXRD patterns of MFM-300(Cr).

**
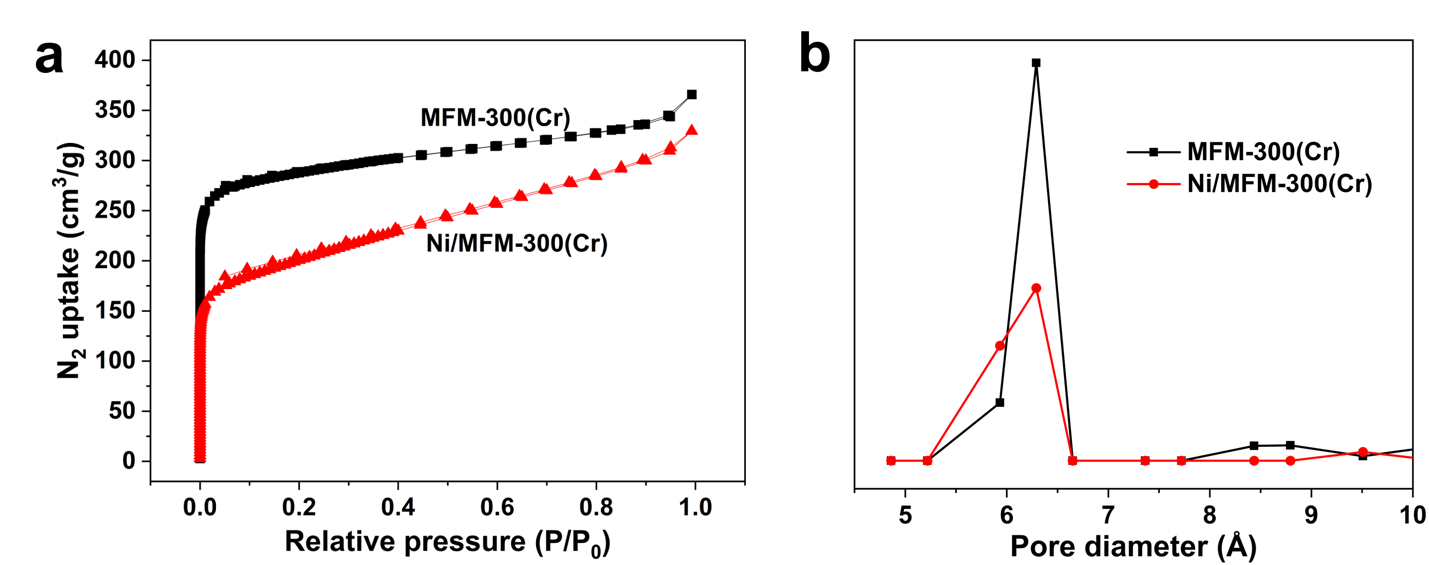
**

**Figure S3:** (a) N_2_ sorption isotherms and (b) pore size distribution of MFM-300(Cr) and 3 wt% Ni/MFM-300(Cr) at 77 K.

**
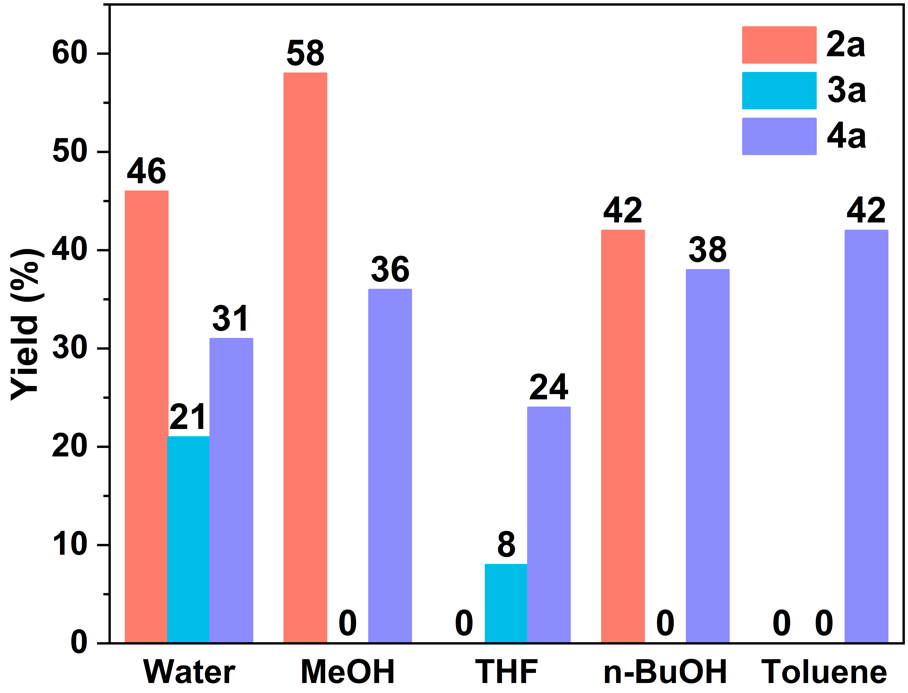
**

**Figure S4: Screening of solvent effects on amination of benzaldehyde as a model substrate.** Reaction conditions: 3 wt% Ni/MFM-300(M) (10mg catalyst, 0.005 mmol Ni), 1 mmol benzaldehyde, NH_3_ in MeOH (1 mL, 7 mmol, 7 mol/L), 1mL MeOH, 5 bar H_2_, 160^o^C, 18 h. Yields were determined by GC using n-dodecane as an internal standard.

**
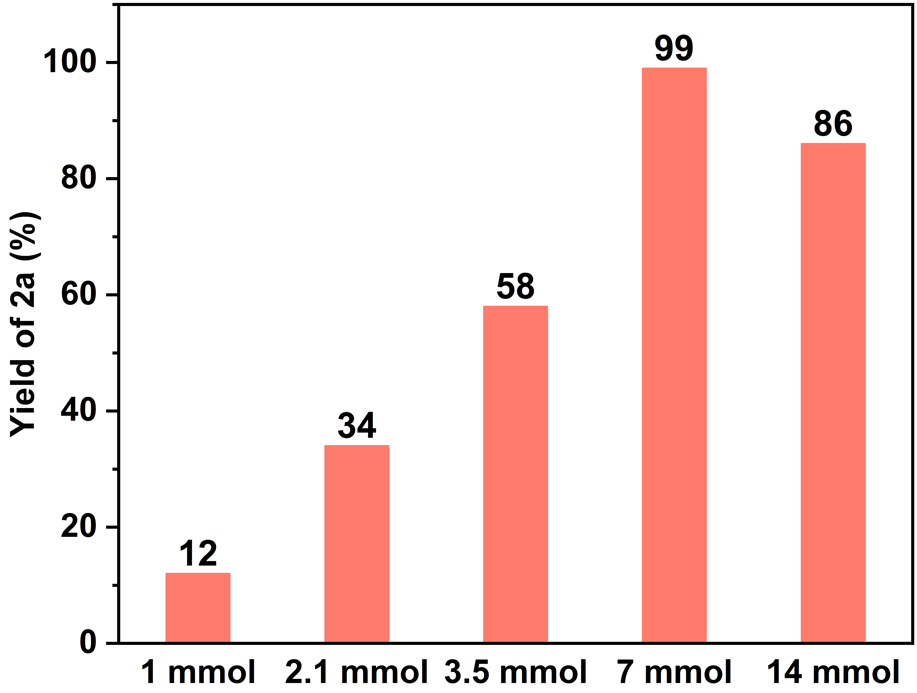
**

**Figure S5: Screening of NH_3_ concentration on the amination of benzaldehyde as a model substrate.** Reaction conditions: 3 wt% Ni/MFM-300(M) (10mg catalyst, 0.005 mmol Ni), 1 mmol benzaldehyde, NH_3_ in MeOH (1 mL), 1mL MeOH, 5 bar H_2_, 160^o^C, 18 h. Yields were determined by GC using n-dodecane as an internal standard.

**
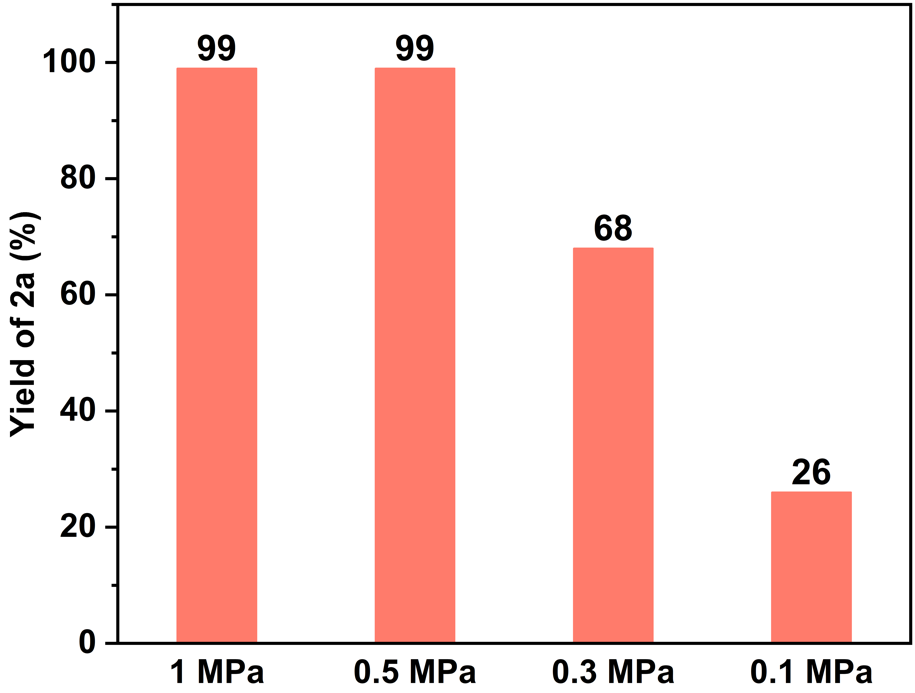
**

**Figure S6: Screening of the effect of H_2_ pressure on amination of benzaldehyde as a model substrate.** Reaction conditions: 3 wt% Ni/MFM-300(M) (10mg catalyst, 0.005 mmol Ni), 1 mmol benzaldehyde, NH_3_ in MeOH (1 mL, 7 mmol, 7 mol/L), 1mL MeOH, 160^o^C, 18 h. Yields were determined by GC using n-dodecane as an internal standard.

**
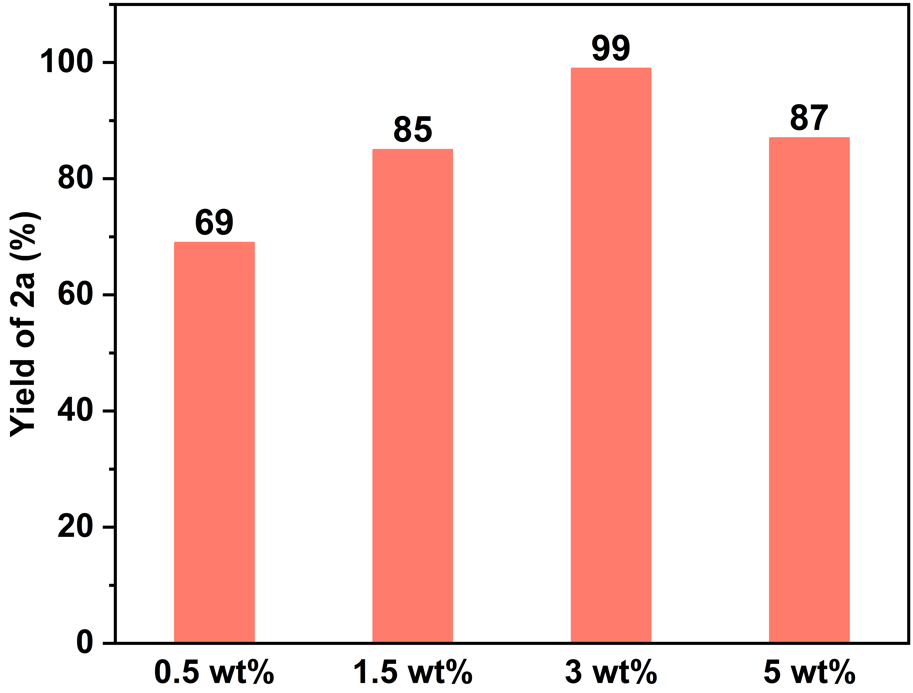
**

**Figure S7: Screening of the effect of metal loading in catalyst on the amination of benzaldehyde as a model substrate.** Reaction conditions: Ni/MFM-300(M) (10mg catalyst), 1 mmol benzaldehyde, NH_3_ in MeOH (1 mL, 7 mmol, 7 mol/L), 1mL MeOH, 5 bar H_2_, 160^o^C, 18 h. Yields were determined by GC using n-dodecane as an internal standard.

**
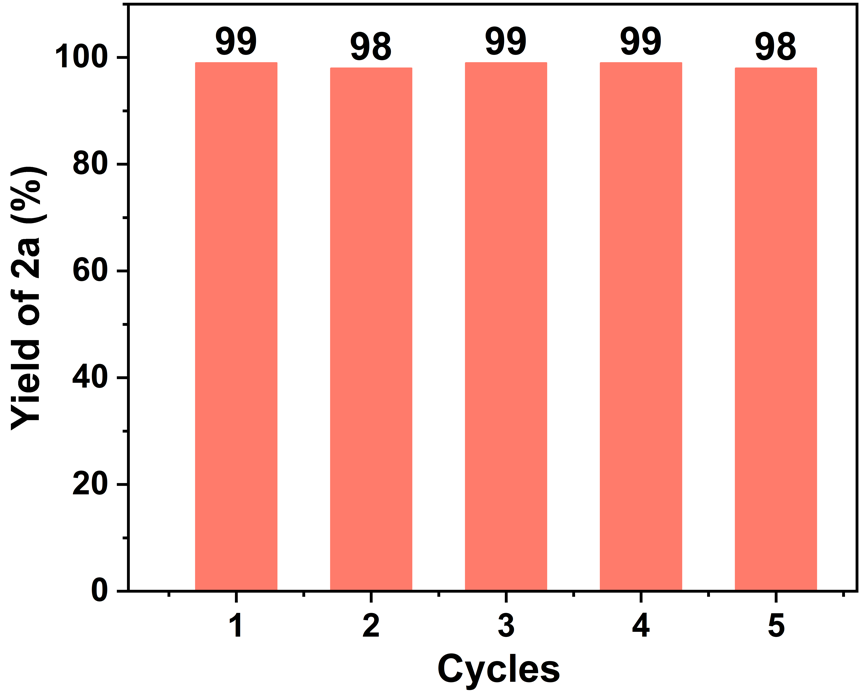
**

**Figure S8: Reusability of Ni/MFM-300(Cr).** The reductive amination of benzaldehyde with NH_3_/H_2_ was chosen to investigate the recyclability of the Ni/MFM-300(Cr) catalyst. For this study, the optimized reaction conditions were used. The yield of benzylamine was determined by GC using n-dodecane as an internal standard. After each run, the catalyst was centrifuged, washed several times with acetone, dried and used directly in the subsequent run. This procedure was done for 5 runs without any decrease in activity.


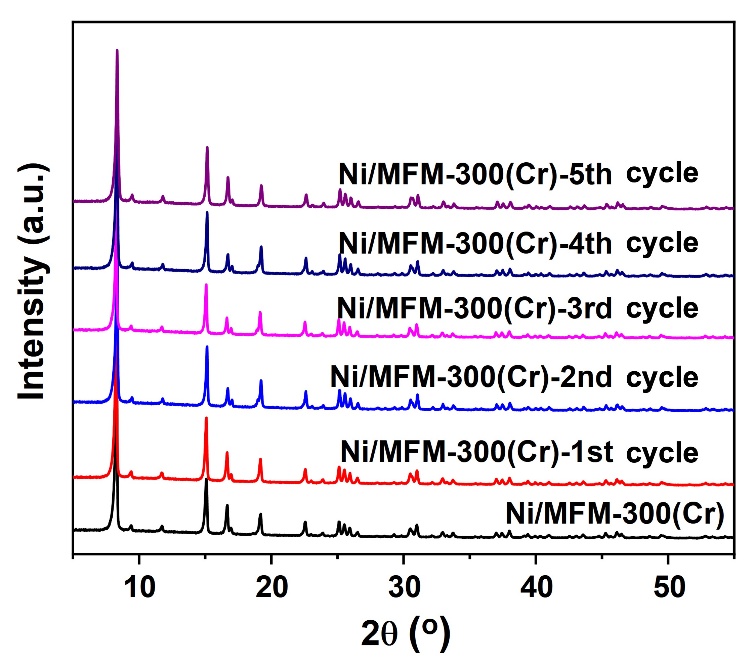


**Figure S9:** PXRD patterns of the fresh and used Ni/MFM-300(Cr) catalyst.

**3. Supplementary Tables**

**Table S1:** Reductive amination of benzyl aldehyde over state-of-the-art catalysts in literature

|  | Catalyst | Temperature/°C | Pressure of H_2_/bar | Metal sites (mmol) | Time (h) | TOF | References |
| --- | --- | --- | --- | --- | --- | --- | --- |
| 1 | 0.5%Ni/MFM-300(Cr) | 160 | 5 | 0.00085 | 18 | 45.1 | This work |
| 2 | 1.5%Ni/MFM-300(Cr) | 160 | 5 | 0.0025 | 18 | 18. 9 | This work |
| 3 | 3%Ni/MFM-300(Cr) | 160 | 5 | 0.005 | 18 | 11.0 | This work |
| 4 | 3%Ni/MFM-300(Cr) | 140 | 5 | 0.005 | 18 | 9.56 | This work |
| 5 | 3%Ni/MFM-300(Cr) | 120 | 5 | 0.005 | 18 | 8.67 | This work |
| 6 | 3%Ni/MFM-300(Cr) | 100 | 5 | 0.005 | 18 | 3.00 | This work |
| 7 | Ni/Al_2_O_3_ | 80 | 10 | 0.006 | 20 | 4.13 | [9] |
| 8 | Ni-TA@SiO_2_-800 | 120 | 20 | 0.03 | 24 | 0.61 | [10] |
| 9 | Ni(BF_4_)_2_6H_2_O + Triphos | 100 | 40 | 0.02 | 24 | 1.01 | [11] |
| 10 | Co-DABCO-TPA@C-800 | 120 | 40 | 0.0175 | 24 | 1.66 | [12] |
| 11 | Co-nanoparticles | 120 | 45 | 0.03 | 24 | 0.62 | [13] |
| 12 | Co@C–N(800) | 35 | 17 | 0.292 | 24 | 0.14 | [14] |
| 13 | Ru_1_/NC-900-800NH_3_ | 100 | 20 | 0.005 | 10 | 38.80 | [15] |
| 14 | Ru/ZrO_2_-150 | 95 | 12 | 0.0495 | 6 | 6.06 | [16] |
| 15 | Ru/TiP-100 | 30 | 17 | 0.002 | 15 | 32.0 | [17] |
| 16 | Ru/Nb_2_O_5_ | 90 | 40 | 0.00123 | 6 | 63.0 | [18] |
| 17 | RuCl_2_(PPh_3_)_3_ | 130 | 40 | 0.01 | 24 | 1.98 | [19] |
| 18 | Fe/(N)SiC | 130 | 65 | 0.05 | 20 | 0.45 | [20] |
| 19 | Ru nanoparticles | 90 | 20 | 0.00198 | 4 | 60.0 | [21] |
| 20 | Co_2_P nanoparticles | 100 | 5 | 0.05 | 10 | 0.94 | [22] |
| 21 | Co/GS@C | 120 | 40 | 0.0175 | 15 | 1.66 | [23] |
| 22 | PtMo WNWs | 80 | 1 | 0.005 | 24 | 0 | [24] |
| 23 | Pt-MoOx/TiO_2_ | 100 | 2 | 0.005 | 20 | 0.90 | [25] |
| 24 | Pt NW | 80 | 1 | 0.005 | 12 | 3.38 | [26] |
| 25 | [Rh(cod)Cl]_2_ | 135 | 65 | 0.0017 | 2 | 8.60 | [27] |

WNWs: worm-like nanowires

NW: nanowire

**Table S2:** Elemental analysis of Ni/support catalysts.

| Samples | Loading amount of Ni (wt%) as measured by ICP-OES |
| --- | --- |
| 0.5wt% Ni/MFM-300(Cr) | 0.48 |
| 1.5wt% Ni/MFM-300(Cr) | 1.53 |
| 3wt% Ni/MFM-300(Cr) | 2.92 |
| 5wt% Ni/MFM-300(Cr) | 4.87 |
| Ni/Cr_2_O_3_ | 2.90 |
| Ni/Al_2_O_3_ | 2.98 |
| Ni/ZrO_2_ | 2.94 |

**Table S3:** Assignment of vibration modes of BPDI

| Simulated | experimental | Vibrational mode |
| --- | --- | --- |
| 218 | 215 | C-C-C twisting |
| 255 | 258 | NH_2_ torsion |
| 285 | 298 | C-C-C twisting |
| 742 | 740 | N-H rocking |
| 773 | 768 | NH_2_ wagging |
| 927 | 933 | N-H wagging of NH_2_ and C-H rocking of CH_2_ |
| 978 | 976 | N-C-N symmetrical stretching |
| 1050 | 1030 | C-N stretching |
| 1106 | 1113 | N-C-N asymmetrical stretching |
| 1184 | 1160 | N_16_-H twisting coupled with C_9_-H rocking |
| 1258 | 1264 | C-N_16_ stretching coupled with C_7_-H, N_16_-H twisting and C-H, N-H rocking |
| 1317 | 1320 | CH_2_ wagging couple with C-H rocking |
| 1361 | 1360 | C_9_-H rocking coupled with NH_2_ twisting |
| 1414 | 1401 | C-H scissoring |

**Table S4:** Assignment of vibration modes of BPI

| Simulated | experimental | Vibrational mode |
| --- | --- | --- |
| 298 | 294 | C-C-C wagging |
| 477 | 477 | C-C-C-C wagging |
| 617 | 620 | Ring deformation |
| 664 | 648 | C-H wagging in benzene ring |
| 740 | 728 | C-H wagging in benzene ring |
| 771 | 777 | C-H wagging in benzene ring |
| 862 | 847 | C-H wagging in benzene ring |
| 911 | 918 | C-H wagging in benzene ring |
| 994 | 990 | C-C bending and C-H rocking in benzene ring and C_9_-H rocking |
| 1045 | 1030 | C-N stretching |
| 1102 | 1102 | C-H rocking |
| 1184 | 1170 | C-H rocking of benzene ring |
| 1255 | 1258 | C_7_-H twisting |
| 1313 | 1317 | C_7_-H wagging |
| 1376 | 1378 | C_9_-H rocking |
| 1481 | 1485 | C-H scissoring |

**Table S5:** Assignment of vibration modes of benzylamine (BA)

| Simulated | experimental | Vibrational mode |
| --- | --- | --- |
| 277 | 266 | NH_2_ torsion |
| 411 | 405 | C-C-C twisting in benzene ring |
| 478 | 464 | C out of plane wagging in benzene ring |
| 586 | 578 | C-H twisting |
| 633 | 623 | C-C wagging in benzene ring |
| 786 | 779 | C-H wagging in benzene ring |
| 833 | 850 | NH_2_ wagging coupled with CH_2_ rocking |
| 925 | 925 | (C-H twisting in benzene ring) |
| 987 | 990 | (C-H twisting in benzene ring) |
| 1053 | 1047 | C-N stretching |
| 1104 | 1120 | C-H rocking in benzene ring |
| 1182 | 1169 | NH_2_ twisting coupled with CH_2_ twisting |
| 1416 | 1436 | C-H wagging coupled with NH_2_ twisting |
| 1482 | 1496 | C-H scissoring |

**3. NMR spectroscopic data**

benzylamine hydrochloride^[22]^

^1^H NMR (500 MHz, DMSO-*d_6_*) δ = 8.78 (bs, 3H), 7.54 (d, *J* = 6.5 Hz, 2H), 7.43-7.26 (m, 3H), 3.99 (s, 2H) ppm.

^13^C NMR (126 MHz, DMSO-*d_6_*) δ = 134.15, 129.12, 128.56, 128.39, 42.17 ppm.

p-tolylmethanamine hydrochloride^[9]^

^1^H NMR (500 MHz, DMSO-*d_6_*) δ = 8.70 (bs, 3H), 7.41 (d, *J* = 7.7 Hz, 2H), 7.17 (d, *J* = 7.8 Hz, 2H), 3.92 (s, 2H), 2.27 (s, 3H) ppm.

^13^C NMR (126 MHz, DMSO-*d_6_*) δ = 137.66, 131.13, 129.08, 129.05, 41.90, 20.83 ppm.

m-tolylmethanamine hydrochloride^[9]^

^1^H NMR (500 MHz, DMSO-*d_6_*) δ = 8.49 (bs, 3H), 7.40-7.24 (m, 3H), 7.19-7.17 (m, 1H), 3.95 (s, 2H), 2.31 (s, 3H) ppm.

^13^C NMR (126 MHz, DMSO-*d_6_*) δ = 137.72, 134.01, 129.51, 128.98, 128.51, 125.98, 42.11, 20.96 ppm.

(3,5-dimethylphenyl)methanamine hydrochloride^[28]^

^1^H NMR (500 MHz, DMSO-*d_6_*) δ = 8.56 (bs, 3H), 7.11 (d, *J* = 1.6 Hz, 2H), 6.99 (s, 1H), 3.90 (s, 2H), 2.27 (s, 6H) ppm.

^13^C NMR (126 MHz, DMSO-*d_6_*) δ = 137.59, 133.96, 129.65, 126.62, 42.04, 20.88 ppm.

(4-ethylphenyl)methanamine hydrochloride^[29]^

^1^H NMR (500 MHz, DMSO-*d_6_*) δ = 8.64 (s, 3H), 7.43 (d, *J* = 7.9 Hz, 2H), 7.22 (d, *J* = 7.8 Hz, 2H), 3.93 (s, 2H), 2.59 (q, *J* = 7.6 Hz, 2H), 1.15 (t, *J* = 7.6 Hz, 3H) ppm.

^13^C NMR (126 MHz, DMSO-*d_6_*) δ = 144.03, 131.40, 129.10, 127.88, 41.87, 27.92, 15.72 ppm.

(4-methoxyphenyl)methanamine hydrochloride^[9]^

^1^H NMR (500 MHz, DMSO-*d_6_*) δ = 8.57 (bs, 3H), 7.44 (d, *J* = 8.7 Hz, 2H), 6.94 (d, *J* = 8.8 Hz, 2H), 3.91 (s, 2H), 3.74 (s, 3H) ppm.

^13^C NMR (126 MHz, DMSO-*d_6_*) δ = 159.32, 130.63, 126.07, 113.89, 55.23, 41.62 ppm.

(4-fluorophenyl)methanamine hydrochloride^[9]^

^1^H NMR (500 MHz, DMSO-*d_6_*) δ = 8.75 (bs, 3H), 7.62-7.57 (m, 2H), 7.23-7.18 (m, 2H), 3.99 (s, 2H) ppm.

^13^C NMR (126 MHz, DMSO-*d_6_*) δ = 162.17 (d, *J* = 244.4 Hz), 131.64 (d, *J* = 7.6 Hz), 130.52 (d, *J* = 3.8 Hz), 115.44 (d, *J* = 21.4 Hz), 41.48 ppm.

1-(4-chlorophenyl)ethanamine hydrochloride^[9]^

^1^H NMR (500 MHz, DMSO-*d_6_*) δ = 8.68 (bs, 3H), 7.56 (d, *J* = 8.5 Hz, 2H), 7.46 (d, *J* = 8.4 Hz, 2H), 3.99 (s, 2H) ppm.

^13^C NMR (126 MHz, DMSO-*d_6_*) δ = 133.17, 133.08, 131.04, 128.46, 41.35 ppm.

(4-bromophenyl)methanamine hydrochloride^[9]^

^1^H NMR (500 MHz, DMSO-*d_6_*) δ = 8.71 (bs, 3H), 7.59 (d, *J* = 8.4 Hz, 2H), 7.49 (d, *J* = 8.5 Hz, 2H), 3.98 (s, 2H) ppm.

^13^C NMR (126 MHz, DMSO-*d_6_*) δ = 133.63, 131.45, 131.43, 121.75, 41.44 ppm.

(4-trifluoromethylphenyl)methanamine hydrochloride^[29]^

^1^H NMR (500 MHz, DMSO-*d_6_*) δ = 8.85 (bs, 3H), 7.78 (d, *J* = 8.3 Hz, 2H), 7.74 (d, *J* = 8.5 Hz, 2H), 4.12 (s, 2H) ppm.

^13^C NMR (126 MHz, DMSO-*d_6_*) δ = 138.90, 129.90, 128.95 (q, *J* = 31.5 Hz), 125.31 (q, *J* = 3.8 Hz), 124.18 (q, *J* = 272.2 Hz), 41.60.

(1,1'-biphenyl)-4-methanamine hydrochloride^[22]^

^1^H NMR (500 MHz, DMSO-*d_6_*) δ = 8.67 (bs, 3H), 7.71-7.67 (m, 4H), 7.62-7.60 (m, 2H), 7.48-7.45 (m, 2H), 7.39-7.36 (m, 1H), 4.05 (s, 2H) ppm.

^13^C NMR (126 MHz, DMSO-*d_6_*) δ = 140.17, 139.55, 133.32, 129.64, 129.00, 127.68, 126.76, 126.70, 41.79 ppm.

Naphthalen-1-ylmethanamine hydrochloride^[30]^

^1^H NMR (500 MHz, DMSO-*d_6_*) δ = 8.83 (bs, 3H), 8.16 (d, *J* = 8.2 Hz, 1H), 8.06-7.92 (m, 2H), 7.70 (d, *J* = 7.0 Hz, 1H), 7.66-7.47 (m, 3H), 4.51 (s, 2H) ppm.

^13^C NMR (126 MHz, DMSO-*d_6_*) δ = 133.20, 130.67, 129.99, 128.98, 128.64, 127.28, 126.74, 126.21, 125.36, 123.51, 39.09 ppm.

Furan-2-ylmethanamine hydrochloride^[22]^

^1^H NMR (400 MHz, DMSO-*d_6_*) δ = 8.77 (bs, 3H), 7.71-7.70 (m, 1H), 6.57-6.56 (m, 1H), 6.47-6.46 (m, 1H), 4.02 (s, 2H) ppm.

^13^C NMR (101 MHz, DMSO-*d_6_*) δ = 147.72, 143.57, 110.98, 110.37, 34.95 ppm.

5-methyl-2-furanylmethanamine hydrochloride^[22]^

^1^H NMR (500 MHz, DMSO-*d_6_*) δ = 8.71 (bs, 3H), 6.42 (d, *J* = 3.1 Hz, 1H), 6.06 (d, *J* = 3.1 Hz, 1H), 3.94 (s, 2H), 2.23 (s, 3H) ppm.

^13^C NMR (126 MHz, DMSO-*d_6_*) δ = 152.13, 145.93, 111.23, 106.98, 34.99, 13.35 ppm.

N,N-dimethylethane-1,2-diamine hydrochloride^[31]^

^1^H NMR (500 MHz, DMSO-*d_6_*) δ = 8.71 (bs, 3H), 3.38 (dd, *J* = 7.7, 6.1 Hz, 2H), 3.24 (t, *J* = 6.8 Hz, 2H), 2.80 (s, 6H) ppm.

^13^C NMR (126 MHz, DMSO-*d_6_*) δ = 53.38, 42.41, 33.65 ppm.

heptan-1-amine hydrochloride^[22]^

^1^H NMR (500 MHz, DMSO-*d_6_*) δ = 7.94 (bs, 3H), 2.51-2.33 (m, 2H), 1.29 (m, 2H), 1.10-0.81 (m, 8H), 0.54 (t, *J* = 6.9 Hz, 3H) ppm.

^13^C NMR (126 MHz, DMSO-*d_6_*) δ = 38.85, 31.21, 28.37, 26.92, 26.06, 22.12, 13.94 ppm.

octan-1-amine hydrochloride^[32]^

^1^H NMR (500 MHz, DMSO-*d_6_*) δ = 8.20 (bs, 3H), 2.76-2.65 (m, 2H), 1.55 (tt, *J* = 7.8, 6.1 Hz, 2H), 1.34-1.14 (m, 10H), 0.83 (t, *J* = 6.8 Hz, 3H) ppm.

^13^C NMR (126 MHz, DMSO-*d_6_*) δ = 38.68, 31.21, 28.56, 28.54, 26.88, 25.95, 22.10, 13.94 ppm.

dodecan-1-amine hydrochloride^[9]^

^1^H NMR (500 MHz, DMSO-*d_6_*) δ = 8.15 (s, 3H), 2.72-2.69 (m, 2H), 1.57-1.51 (m, 2H), 1.28-1.22 (m, 18H), 0.85-0.82 (m, 3H) ppm.

^13^C NMR (126 MHz, DMSO-*d_6_*) δ = 38.68, 31.33, 29.08, 29.06, 28.99, 28.89, 28.75, 28.60, 26.92, 25.92, 22.12, 13.96 ppm.

3-phenylpropan-1-amine hydrochloride^[12]^

^1^H NMR (500 MHz, DMSO-*d_6_*) δ = 8.36 (bs, 3H), 7.25 (t, *J* = 7.5 Hz, 2H), 7.21-7.08 (m, 3H), 2.82-2.70 (m, 2H), 2.62 (t, *J* = 7.8 Hz, 2H), 1.89 (p, *J* = 7.6 Hz, 2H) ppm.

^13^C NMR (126 MHz, DMSO-*d_6_*) δ = 140.97, 128.44, 128.33, 126.03, 38.37, 31.98, 28.71 ppm.

1-phenylethanamine hydrochloride^[9]^

^1^H NMR (500 MHz, DMSO-*d_6_*) δ = 8.75 (bs, 3H), 7.59-7.52 (m, 2H), 7.43-7.33 (m, 3H), 4.38-4.34 (m, 1H), 1.53 (d, *J* = 6.8 Hz, 3H) ppm.

^13^C NMR (126 MHz, DMSO-*d_6_*) δ = 139.54, 128.65, 128.30, 126.91, 50.05, 20.90 ppm.

1-(p-tolyl)ethanamine hydrochloride^[9]^

^1^H NMR (500 MHz, DMSO-*d_6_*) δ = 8.66 (bs, 3H), 7.42 (d, *J* = 8.2 Hz, 2H), 7.21 (d, *J* = 7.9 Hz, 2H), 4.33-4.29 (m, 1H), 2.30 (s, 3H), 1.50 (d, *J* = 6.8 Hz, 3H) ppm.

^13^C NMR (126 MHz, DMSO-*d_6_*) δ = 137.59, 136.52, 129.12, 126.80, 49.75, 20.81, 20.71 ppm.

1-(3,4-dimethylphenyl)ethanamine hydrochloride^[9]^

^1^H NMR (500 MHz, DMSO-*d_6_*) δ = δ 8.66 (bs, 3H), 7.30 (s, 1H), 7.23-7.25 (m, 1H), 7.14 (d, *J* = 7.7 Hz, 1H), 4.25 (m, 1H), 2.21 (d, *J* = 6.8 Hz, 6H), 1.49 (d, *J* = 6.8 Hz, 3H) ppm.

^13^C NMR (126 MHz, DMSO-*d_6_*) δ = 136.87, 136.34, 136.25, 129.61, 127.97, 124.16, 49.80, 20.85, 19.48, 19.06 ppm.

1-(4-methoxyphenyl)ethanamine hydrochloride^[9]^

^1^H NMR (500 MHz, DMSO-*d_6_*) δ = 8.64 (bs, 3H), 7.47 (d, *J* = 8.8 Hz, 2H), 6.95 (d, *J* = 8.8 Hz, 2H), 4.30 (p, *J* = 6.0 Hz, 1H), 3.74 (s, 3H), 1.50 (d, *J* = 6.8 Hz, 3H) ppm.

^13^C NMR (126 MHz, DMSO-*d_6_*) δ = 159.18, 131.42, 128.33, 113.94, 55.22, 49.51, 20.78 ppm.

1-(3-methoxyphenyl)ethan-1-amine hydrochloride

^1^H NMR (500 MHz, DMSO-*d_6_*) δ = δ 8.74 (bs, 3H), 7.30 (t, *J* = 7.9 Hz, 1H), 7.22 (t, *J* = 2.2 Hz, 1H), 7.09 (dt, *J* = 7.8, 1.2 Hz, 1H), 6.90 (ddd, *J* = 8.3, 2.6, 0.9 Hz, 1H), 4.32 (q, *J* = 6.8 Hz, 1H), 3.76 (s, 3H), 1.51 (d, *J* = 6.8 Hz, 3H) ppm.

^13^C NMR (126 MHz, DMSO-*d_6_*) δ = 159.45, 141.13, 129.78, 118.93, 113.79, 112.63, 55.28, 50.07, 20.93 ppm.

1-(phenyl)propylamine hydrochloride

^1^H NMR (500 MHz, DMSO-*d_6_*) δ = 8.84 (bs, 3H), 7.55 (d, *J* = 7.3 Hz, 2H), 7.39-7.31 (m, 3H), 4.09-4.07 (m, 1H), 2.09-2.02 (m, 1H), 1.87-1.79 (m, 1H), 0.72-0.69 (m, 3H) ppm.

^13^C NMR (126 MHz, DMSO-*d_6_*) δ = 137.74, 128.54, 128.32, 127.61, 55.92, 27.47, 10.03 ppm.

Cyclopentanamine hydrochloride^[33]^

^1^H NMR (500 MHz, DMSO-*d_6_*) δ = 8.27 (s, 3H), 3.43-3.38 (m, 1H), 1.89-1.82 (m, 2H), 1.73-1.65 (m, 2H), 1.62-1.54 (m, 2H), 1.53-1.45 (m, 2H) ppm.

^13^C NMR (126 MHz, DMSO-*d_6_*) δ = 51.18, 30.58, 23.44 ppm.

cyclohexanamine hydrochloride^[22]^

^1^H NMR (500 MHz, DMSO-*d_6_*) δ = 8.24 (s, 3H), 2.92-2.86 (m, 1H), 1.94-1.90 (m, 2H), 1.71-1.66 (m, 2H), 1.57-1.53 (m, 1H), 1.34-1.17 (m, 4H), 1.10-1.02 (m, 1H) ppm.

^13^C NMR (126 MHz, DMSO-*d_6_*) δ = 49.29, 30.22, 24.64, 23.77 ppm.

Cycloheptanamine hydrochloride

^1^H NMR (400 MHz, DMSO-*d_6_*) δ = 8.22 (s, 3H), 3.11-3.04 (m, 1H), 1.98-1.91 (m, 2H), 1.66-1.58 (m, 2H), 1.57-1.26 (m, 8H) ppm.

^13^C NMR (126 MHz, DMSO-*d_6_*) δ = 51.61, 32.27, 27.31, 23.08 ppm.

pentan-3-amine hydrochloride

^1^H NMR (500 MHz, DMSO-*d_6_*) δ = 8.22 (s, 3H), 2.89-2.84 (m, 1H), 1.60-1.51 (m, 4H), 0.86 (t, *J* = 7.6 Hz, 6H) ppm.

^13^C NMR (126 MHz, DMSO-*d_6_*) δ = 53.20, 24.18, 9.42 ppm.

**
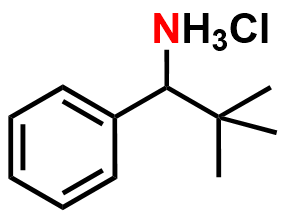
**

**2,2-dimethyl-1-phenylpropan-1-amine hydrochloride**

^1^H NMR (400 MHz, DMSO-*d_6_*) δ = 8.61 (s, 3H), 7.46-7.34 (m, 5H), 4.07 (s, 1H), 0.94 (s, 9H) ppm.

^13^C NMR (101 MHz, DMSO-*d_6_*) δ = 136.02, 128.45, 128.12, 127.92, 63.22, 33.92, 26.28 ppm.


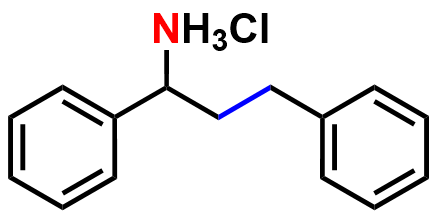


**1,3-diphenylpropan-1-amine hydrochloride**

^1^H NMR (400 MHz, DMSO-*d_6_*) δ = 8.78 (s, 3H), 7.57-7.54 (m, 2H), 7.47-7.37 (m, 3H), 7.29-7.25 (m, 2H), 7.20-7.11 (m, 3H), 4.18 (dd, *J* = 8.0, 4.0 Hz, 1H), 2.56-2.53 (m, 1H), 2.47-2.29 (m, 2H), 2.19-2.08 (m, 1H).

^13^C NMR (101 MHz, DMSO-*d_6_*) δ = 140.62, 137.70, 128.75, 128.58, 128.42, 128.13, 127.61, 126.03, 54.20, 35.92, 31.14 ppm.


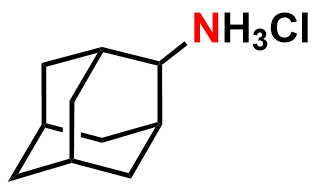


**(1r,3r,5r,7r)-adamantan-2-amine hydrochloride**

^1^H NMR (400 MHz, DMSO-*d_6_*) δ = 8.31 (s, 3H), 3.26 (s, 1H), 2.07-1.99 (m, 4H), 1.83-1.68 (m, 8H), 1.54-1.50 (m, 2H).

^13^C NMR (101 MHz, DMSO-*d_6_*) δ = 54.63, 36.82, 36.12, 29.94, 29.46, 26.39, 26.30 ppm.

**4. NMR spectra**

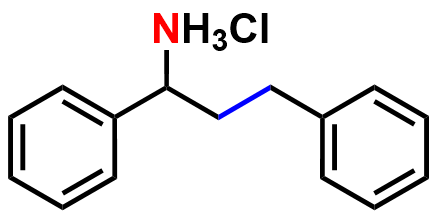


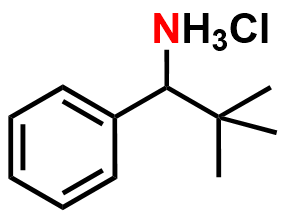


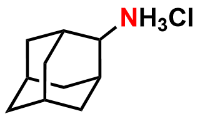


**Reference**

1. T. Luo, L. Li, Y. Chen, J. An, C. Liu, Z. Yan, J. H. Carter, X. Han, A. M. Sheveleva, F. Tuna, E. J. L. McInnes, C. C. Tang, M. Schröder, S. Yang, *Nat. Commun.* **2021**, *12*, 3583.

2. Q. Zhu, J. Li, Q. Xu, *J. Am. Chem. Soc.* **2013**, *135*, 10210-10213.

3. S. J. Clark, M. D. Segall, C. J. Pickard, P. J. Hasnip, M. I. J. Probert, K. Refson, M. C. Payne, *Z. Krist-Cryst. Mater*. **2005**, *220*, 567-570.

4. K. Refson, P. R. Tulip, S. J. Clark, *Phys. Rev. B—Condens. Matter Mater. Phys.* **2006**, *73*, 155114.

5. J. P. Perdew, K. Burke, M. Ernzerhof, *Phys. Rev. Lett.* **1996**, *77*, 3865.

6. E.R. McNellis, J. Meyer, K. Reuter, *Phys. Rev. B—Condens. Matter Mater. Phys.* **2009**, *80*, 205414.

7. Y. Cheng, L. Daemen, A. Kolesnikov, A. J. Ramirez-Cuesta, *J. Chem. Theory Comput.* **2019**, *15*, 1974-1982.

8. A. J. Ramirez-Cuesta, Comput. Phys. Commun. **2004**, *157*, 226-238.

9. G. Hahn, P. Kunnas, N. de Jonge, R. Kempe, *Nat. Catal.* **2019**, *2*, 71-77.

10. K. Murugesan, M. Beller, R. V. Jagadeesh, *Angew. Chem. Int. Ed.* **2019**, *131*, 5118-5122.

11. K. Murugesan, Z. Wei, V. G. Chandrashekhar, H. Jiao, M. Beller, R. V. Jagadeesh, *Chem. Sci.* **2020**, *11*, 4332-4339.

12. R. V. Jagadeesh, K. Murugesan, A. S. Alshammari, H. Neumann, M. M. Pohl, J. Radnik, M. Beller, *Science* **2017**, *358*, 326-332.

13. T. Senthamarai, V. G. Chandrashekhar, M. B. Gawande, N. V. Kalevaru, R. Zbořil, P. C. J. Kamer, R. V. Jagadeesh, M. Beller, *Chem. Sci.* **2020**, *11*, 2973-2981.

14. B. Zheng, J. Xu, J. Song, H. Wu, X. Mei, K. Zhang, W. Han, W. Wu, M. He, B. Han, *Chem. Sci.* **2022**, *13*, 9047-9055.

15. H. Qi, J. Yang, F. Liu, L. Zhang, J. Yang, X. Liu, L. Li, Y. Su, Y. Liu, R. Hao, A. Wang, T. Zhang, *Nat. Commun.* **2021**, *12*, 3295.

16. G. Liang, A. Wang, L. Li, G. Xu, N. Yan, T. Zhang, *Angew. Chem. Int. Ed.* **2017**, *56*, 3050-3054.

17. C. Xie, J. Song, M. Hua, X. Huang, H. Wu, G. Yang, B. Han, *ACS Catal*. **2020**, *10*, 7763-7772.

18. T. Komanoya, T. Kinemura, Y. Kita, K. Kamata, M. Hara, *J. Am. Chem. Soc.* **2017**, *139*, 11493-11499.

19. T. Senthamarai, K. Murugesan, J. Schneidewind, N. V. Kalevaru, W. Baumann, H. Neumann, P. C. J. Kamer, M. Beller, R. V. Jagadeesh, *Nat. Commun.* **2018**, *9*, 4123.

20. C. Bäumler, C. Bauer, R. Kempe, *ChemSusChem*, **2020**, *13*, 3110-3114.

21. D. Chandra, Y. Inoue, M. Sasase, M. Kitano, A. Bhaumik, K. Kamata, H. Hosono, M. Hara, *Chem. Sci.* **2018**, *9*, 5949-5956.

22. M. Sheng, S. Fujita, S. Yamaguchi, J. Yamasaki, K. Nakajima, S. Yamazoe, T. Mizugaki, T. Mitsudome, *JACS Au*, **2021**, *1*, 501-507.

23. K. Murugesan, V. G. Chandrashekhar, T. Senthamarai, R. V. Jagadeesh, M. Beller, *Nat. Protoc.* **2020**, *15*, 1313-1337.

24. S. Lu, P. Xu, X. Cao, H. Gu, RSC Adv. **2018**, *8*, 8755-8760.

25. Y. Nakamura, K. Kon, A. S. Touchy, K. Shimizu, W. Ueda, *ChemCatChem*, **2015**, *7*, 921-924.

26. F. Qi, L. Hu, S. Lu, X. Cao, H. Gu, *Chem. Commun.* **2012**, *48*, 9631-9633.

27. T. Gross, A. M. Seayad, M. Ahmad, M. Beller, *Org. Lett.* **2002**, *4*, 2055-2058.

28. E. A. Meade, M. Sznaidman, G. T. Pollard, L. M. Beauchamp, J. L. Howard, *Eur. J. Med. Chem*. **1998**, *33*, 363-374.

29. N. Gandhamsetty, J. Jeong, J. Park, S. Park, S. Chang, *J. Org. Chem.* **2015**, *80*, 7281-7287.

30. W. Yao, H. Fang, Q. He, D. Peng, G. Liu, Z. Huang, *J. Org. Chem.* **2019**, *84*, 6084-6093.

31. Y. Zang, Q. Sui, Q. Xu, M. Ma, G. Li, F. Zhu, *Tetrahedron Lett.* **2023**, *124*, 154598.

32. A. Sanagawa, H. Nagashima, *Org. Lett.* **2018**, *21*, 287-291.

33. D. Cantillo, M. M. Moghaddam, C. O. Kappe, *J. Org. Chem.* **2013**, *78*, 4530-4542.
